# Trapped in Endosome PEGylated Ultra‐Small Iron Oxide Nanoparticles Enable Extraordinarily High MR Imaging Contrast for Hepatocellular Carcinomas

**DOI:** 10.1002/advs.202401351

**Published:** 2024-08-20

**Authors:** Dandan Zhou, Shanshan Shan, Lei Chen, Cang Li, Hongzhao Wang, Kuan Lu, Jianxian Ge, Ning Wang, Mohammad Javad Afshari, Yaqin Zhang, Jianfeng Zeng, Mingyuan Gao

**Affiliations:** ^1^ Center for Molecular Imaging and Nuclear Medicine State Key Laboratory of Radiation Medicine and Protection School for Radiological and Interdisciplinary Sciences (RAD‐X) Soochow University Collaborative Innovation Center of Radiological Medicine of Jiangsu Higher Education Institutions Suzhou 215123 P. R. China; ^2^ Department of Radiology The Fifth Affiliated Hospital of Sun Yat‐sen University Zhuhai 519000 P. R. China

**Keywords:** contrast agent, early diagnosis, hepatocellular carcinoma, iron oxide nanoparticle

## Abstract

The early diagnosis of hepatocellular carcinomas (HCCs) remains challenging in the clinic. Primovist‐enhanced magnetic resonance imaging (MRI) aids HCC diagnosis but loses sensitivity for tumors <2 cm. Therefore, developing advanced MRI contrast agents is imperative for improving the diagnostic accuracy of HCCs in very‐early‐stage. To address this challenge, PEGylated ultra‐small iron oxide nanoparticles (PUSIONPs) are synthesized and employed as liver‐specific T1 MRI contrast agents. Intravenous delivery produces simultaneous hyperintense HCC and hypointense hepatic parenchyma signals on T1 imaging, creating an extraordinarily high tumor‐to‐liver contrast. Systematic studies uncover PUSIONP distribution in hepatic parenchyma, HCC lesions at the organ, tissue, cellular, and subcellular levels, revealing endosomal confinement of PUSIONP without aggregation. By mimicking such situations, the dependency of relaxometric properties on local PUSIONP concentration is investigated, emphasizing the key role of different endosomal concentrations in liver and tumor cells for high tumor‐to‐liver contrast and clear tumor boundaries. These findings offer exceptional imaging capabilities for early HCC diagnosis, potentially benefiting real HCC patients.

## Graphical abstract

PEGylated ultra‐small iron oxide nanoparticles (PUSIONPs) enhance MRI contrast for hepatocellular carcinoma detection by exploiting the concentration differences between tumors and liver tissues. This differential accumulation in endosomes of tumor and liver cells leads to simultaneous hyperintense tumor and hypointense liver signals, providing high tumor‐to‐liver contrast for early‐stage HCC diagnosis.

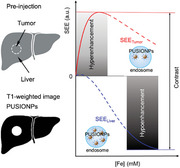

## 1 Introduction

Hepatocellular carcinoma (HCC) is the most common type of primary liver cancer that has dropped very recently from second to third cancer‐related deaths.^[^ ^1^ ^]^ As timely treatment can increase the 5‐year survival rate of HCC patients from 3% to above 50%,^[^ ^2^ ^]^ a precise diagnosis of early HCC therefore becomes one of the most sought‐after desires, through which the therapeutic efficacy and overall survival rate are expected to be further improved.^[^ ^2^, ^3^ ^]^

Thus far, HCCs are mainly detected through regular serology or ultrasound screening.^[^ ^4^ ^]^ In cases where an elevation in the level of α‐fetoprotein or suspicious lesions are reported, dynamic imaging techniques such as contrast‐enhanced magnetic resonance imaging (CE‐MRI) or contrast‐enhanced computerized tomography (CE‐CT) are usually employed for the further diagnosis. In comparison with CE‐CT, CE‐MRI exhibits higher diagnostic specificity and sensitivity owing to superior soft tissue contrast and excellent submillimeter spatial resolution.^[^ ^2^, ^5^ ^]^ Nevertheless, MRI contrast agents are indispensable. Currently, the clinical MRI contrast agents for HCC diagnoses are chosen from gadolinium complexes, which can further be divided into two categories, i.e., nonspecific contrast agents, e.g., Gd‐DTPA, Magnevist and liver‐specific contrast agents, e.g., Gd‐EOB‐DTPA, Primovist.^[^ ^4^, ^5^, ^6^ ^]^ HCC imaging by nonspecific MRI contrast agents features typical hyperenhancement in the arterial phase followed by isoenhancement/hypoenhancement in the venous/delayed phase.^[^ ^6a^ ^]^ Such dynamic MR imaging offers a diagnostic sensitivity and specificity of up to 90% and 95%, respectively, according to clinical studies.^[^ ^5^ ^]^ However, the dynamic behaviors of HCCs are significantly dependent on the tumor size and differentiation status. For example, the sensitivity is dramatically decreased to 62% for HCCs smaller than 2 cm,^[^ ^7^ ^]^ owing to their immature arterialization during hepatocarcinogenesis.^[^ ^8^ ^]^ In contrast, the liver‐specific contrast agent improves the diagnostic sensitivity of small HCC by offering an additional hepatobiliary phase induced by selective uptake of the contrast agent by hepatocytes in the noncancerous hepatic tissue, apart from the contrast enhancement pattern similar to that for the nonspecific contrast agents, thus providing more precise diagnoses on small HCCs.^[^ ^7^, ^8^ ^]^ For example, Primovist can improve the specificity to almost 100%.^[^ ^9^ ^]^ Nevertheless, the sensitivity for small cirrhosis nodules of 1–2 cm remains ≈70% and apparently needs to be improved.^[^ ^2^, ^9^ ^]^ Moreover, it is very challenging to reliably diagnose liver nodules <1 cm.^[^ ^2d^ ^]^ Thus, a close follow‐up has to be carried out for screening early HCCs but is often ignored by patients.^[^ ^10^ ^]^ Therefore, developing advanced MRI contrast agents becomes imperative for improving the diagnostic accuracy of HCCs in the very‐early‐stage.

Ferumoxides based iron oxide nanoparticles (IONPs) were developed as liver‐specific MRI contrast agents and got approved by the FDA in 1996. Different from gadolinium complexes used for T1‐weighted MRI, ferumoxides were used as T2 contrast agents in the clinics. They are specifically phagocytosed by the reticuloendothelial system and provide the liver parenchyma with a hypointense signal,^[^ ^11^ ^]^ thus offering a high contrast to the liver lesions that show limited capability to uptake IONPs. However, the imaging characteristics of HCCs are often influenced by inflammation and fat.^[^ ^12^ ^]^ Moreover, it is difficult to distinguish HCCs from benign lesions, e.g., hemangiomas and cysts, through contrast‐enhanced T2‐weighted MR imaging.^[^ ^3^, ^13^ ^]^ Although Feridex offers no obvious advantages over Magnevist in detecting small HCCs (≤1.5 cm),^[^ ^14^ ^]^ numerous studies indicate that IONPs may have remarkable potential owing to the rich relaxometric properties derived from the delicate combinations of particle size, surface chemistry, and even stimuli‐responsive assembly/disassembly.^[^ ^13^, ^15^ ^]^ This potential has prompted extensive exploration of IONPs ‐based nanomaterials for various applications, including tumor detection,^[^ ^16^ ^]^ magnetic resonance angiography,^[^ ^17^ ^]^ and cerebral MRI.^[^ ^18^ ^]^ Typically, reducing the diameter of IONPs to less than 5 nm leads to a strong T1 effect, while the T2 effect is weakened, which switches the IONPs from a typical T2 contrast agent to a T1 or T1/T2 dual‐modality contrast agent.^[^ ^11^, ^19^ ^]^ Additionally, the surface chemistry of IONPs, such as the inclusion of anchoring groups with conjugated structures, can enhance local magnetic field inhomogeneity around the IONPs, thereby boosting T2 imaging performance.^[^ ^20^ ^]^ Furthermore, changing the aggregation status can remarkably alter the relaxation properties of IONPs, providing a unique opportunity to design T1/T2‐switchable contrast agents to improve the accuracy and sensitivity of HCC diagnosis.^[^ ^2^, ^12^, ^21^ ^]^ For example, Lu et al. designed an i‐motif DNA‐assisted pH‐responsive iron oxide nanocluster assembly that can be disassembled in response to the acidic tumor microenvironment to brighten HCC lesions on T1‐weighted MRI, while the assemblies captured by hepatic parenchyma remain integral to darken the normal liver on T1‐weighted imaging.^[^ ^2^, ^22^ ^]^ These inverse contrast‐enhancing tendencies for normal liver tissue and HCC lesions are apparently favorable for improving the diagnostic sensitivity of HCCs. Furthermore, Zhang et al. reported ultra‐small BSA‐modified IONPs as T1/T2 dual‐modality MRI contrast agents.^[^ ^12^ ^]^ Upon intravenous administration, the hepatic parenchyma rapidly darkened on T2‐weighted imaging, while the tumorous site gradually brightened on T1‐weighted imaging. It was explained in a way that the hepatic parenchyma quickly phagocytized the nanoparticles that aggregated to induce T2 contrast enhancement, while the nanoparticles gradually accumulated in the tumor through enhanced permeation and retention effects. Due to the low concentration in the latter situation, the nanoparticles remained disperse to give rise to T1 contrast enhancement. If the hypoenhancement of benign liver tissue and hyperenhancement of HCCs are simultaneously achieved through single modality imaging, a more precise diagnosis of HCCs can be expected. However, there is no systematic study on such imaging patterns ever reported before. Moreover, the complicated interactions between nano‐contrast agents and various types of cells in the liver and their impacts on contrast‐enhancing patterns are far from understood.

To face the challenge of early HCC diagnoses and address the related fundamental questions regarding nano‐contrast agents, we report PEGylated ultra‐small IONPs (PUSIONPs) with simple composition and structure as liver‐specific MRI contrast agents for the highly sensitive diagnosis of tiny HCCs. The as‐prepared PUSIONPs effectively darkened the hepatic parenchyma and simultaneously brightened the HCC lesions on the T1‐weighted MRI. To discover the underlying mechanism, the distributions of PUSIONPs in hepatic parenchyma and HCC lesions were systematically studied at the organ, tissue, cellular, and even subcellular levels. A difference in the enrichment degree of PUSIONPs in the endosomes of liver cells and tumor cells was observed and investigated experimentally and theoretically to disclose the underlying mechanisms leading to the inverse variations in MRI signals from hepatic parenchyma and HCC lesions, respectively. Moreover, the imaging capacity of PUSIONPs was also evaluated by integrating HCC patient‐derived imaging information into the theoretical framework for contrast enhancement efficacy to show the potential for clinical applications.

## 2 Results

### 2.1 PUSIONPs Synthesis and Characterization

**Figure 1:**
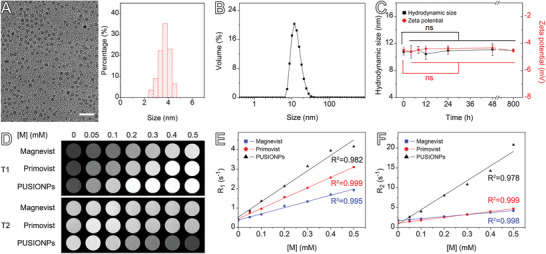
Characterization of PUSIONPs. A) TEM image and size distribution histogram of hydrophilic iron oxide nanoparticles (the scale bar corresponds to 20 nm). B) Hydrodynamic size profile of PUSIONPs. C) Temporal evolution of the hydrodynamic size and zeta potential of PUSIONPs in water. Data are presented as mean ± SD, *n* = 3, with *p*‐values calculated using unpaired Student's *t*‐test. The mean values at all other time points were not statistically different from the mean values at 0 h (*p* > 0.05). D) T1‐ and T2‐weighted images of aqueous solutions containing different concentrations of Magnevist, Primovist, and PUSIONPs. E,F) Iron concentration‐dependent R_1_ (E) and R_2_ (F) for extracting longitudinal and transverse relaxivities through linear regression fitting.

The hydrophobic ultra‐small IONPs with an average size of 3.6 nm, as shown in Figure S1 (Supporting Information), were synthesized upon thermal decomposition of the iron precursor through flow synthesis.^[^ ^23^ ^]^ By replacing the native oleate ligands with polyethylene glycol bearing a diphosphonate and a methoxyl group on different sides (DP‐PEG), hydrophilic PUSIONPs were obtained, as shown in **Figure** **1**A. The magnetization curve of PUSIONPs, presented in Figure S2 (Supporting Information), indicates that the PUSIONPs exhibit superparamagnetic behavior. The dynamic light scattering (DLS) profile in Figure 1B reveals that the as‐prepared water‐soluble PUSIONPs have a hydrodynamic size of 11.7 nm. Negligible variations in the hydrodynamic size and zeta potential over 1 month demonstrate that the resulting PUSIONPs are rather colloidally stable in aqueous solution (Figure 1C), owing to the firm attachment of the biocompatible DP‐PEG ligands on the particle surface, which is favorable for further in vivo applications.

The MRI contrast enhancement effects of PUSIONPs were evaluated on a preclinical 3T MRI scanner. Figure 1D shows T1‐ and T2‐weighted images of aqueous solutions containing different concentrations of Magnevist, Primovist, and PUSIONPs. In comparison with clinical gadolinium‐based contrast agents, PUSIONPs exhibit significantly improved T1 and T2 contrast performances. The quantitative analyses shown in Figure 1E,F reveal that the longitudinal relaxivity (*r*_1_) and transverse relaxivity (*r*_2_) obtained upon linear regression fitting of the experimental data were 3.2 and 5.1 mm ^−1^ s^−1^ for Magnevist, 5.3 and 7.2 mm ^−1^ s^−1^ for Primovist, and 7.9 and 36.7 mm ^−1^ s^−1^ for PUSIONPs, respectively.

### 2.2 In Vivo MR Imaging with PUSIONPs

**Figure 2:**
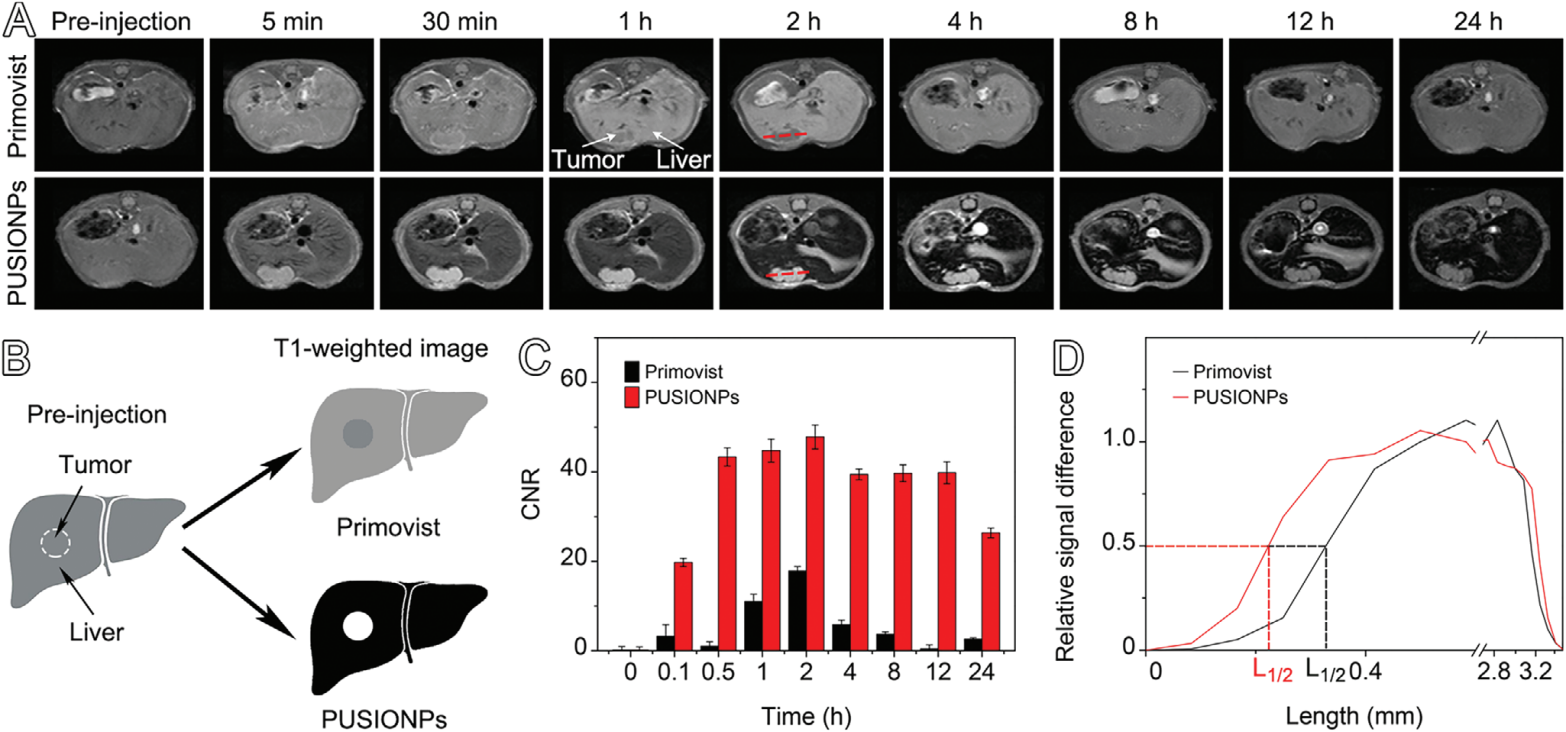
MRI of mice bearing orthotopic HCC xenografts with PUSIONPs. A) T1‐weighted MR images acquired with the FSE sequence at different time points after intravenous injection of Primovist and PUSIONPs. B) Enhancement patterns showing the advantage of PUSIONPs over Primovist on T1‐weighted MRI of HCCs shown as a round area in the liver. C) Temporal variations in CNR obtained with Primovist and PUSIONPs. Data are presented as mean ± SD, *n* = 1. The mean represents the signal intensity in the region of interest (ROI), while the SD denotes the standard deviation calculated from the ROI of MR images. D) Profiles of the relative signal drawn along the red lines in the MRI images in panel A.

To assess the capacity of PUSIONPs for detecting HCCs in vivo, BALB/c nude mice bearing orthotopic HCC xenografts of ≈15 mm^3^ were adopted for the following imaging studies. To ensure that the imaging results were comparable, contrast‐enhanced imaging was performed on the same group of tumor‐bearing mice in such a way that the administration of Primovist was followed by PUSIONPs, but with an interval of 24 h between the two shots. It is worth mentioning that the interference of Primovist on PUSIONPs can be neglected owing to the rapid excretion of the former agent.^[^ ^6^, ^7^ ^]^ T1‐weighted MR images acquired with the FSE sequence after intravenous injection of Primovist (0.10 mmol Gd/kg of body weight) are shown in the upper panel of **Figure** **2**A. The HCC lesion is hardly recognized pre‐contrasted. Owing to the specific uptake of Primovist by hepatocytes, the benign liver region is gradually brightened. In contrast, the hepatic parenchyma darkened after administration of PUSIONPs (0.10 mmol Fe/kg of body weight), meanwhile the HCC lesion brightened on T1‐weighted MR imaging, as shown in the lower panel of Figure 2A, which remarkably increased the tumor‐to‐liver contrast.

Careful comparison revealed that the liver and tumor exhibited similar signal variations after the administration of Primovist, characterized by a rapid signal increase followed by a gradual decline, as shown in Figure S3 (Supporting Information). However, PUSIONPs gave rise to different signal‐varying trends for benign liver and tumor tissues. Although the tumor site also shows a hyperintense signal that declined over time, the hepatic parenchyma displays all the way hypointense signals, as shown in the lower panel of Figure 2A. Thus, PUSIONPs offer a much higher imaging contrast than Primovist, as schematically shown in Figure 2B. To quantify the contrast enhancement effects, a contrast‐to‐noise ratio (CNR) between the tumor and the surrounding liver tissue was calculated. As presented in Figure 2C, the CNRs for both PUSIONPs and Primovist keep increasing to reach their corresponding maximum values at ≈2 h. However, the maximum value for PUSIONPs is much higher than that for Primovist, i.e., 47.8 versus 17.9, demonstrating that PUSIONPs are much more sensitive than Primovist in the HCC diagnosis. This superiority not only persists but also gets amplified as time elapses due to the faster decline of the Primovist signal from the tumor. In addition, the boundaries delineated by PUSIONPs are much sharper than those obtained with Primovist, as shown in Figure 2A. If the distance to reach half of the maximum CNR value (termed as L_1/2_) is used to quantitatively describe the clarity of the tumor boundaries, as shown in Figure 2D, the L_1/2_ along the red line across the tumor in Figure 2A is shortened by 24.2% when comparing PUSIONPs with Primovist. All these results demonstrate that PUSIONPs hold great potential as a high‐performance liver‐specific contrast agent that provides not only higher sensitivity and a longer imaging window for HCC diagnosis but also improved clarity of tumor boundaries. Nevertheless, the mechanism behind the extraordinary tumor‐to‐liver contrast remains far from understood.

### 2.3 Understanding MRI Signal Enhancement Efficiency

Generally, the MRI signal intensity (SINT) depends on many factors, including imaging parameters, tissue topology, and contrast agent concentration. The SINT in the FSE sequence can be given by:^[^ ^24^ ^]^

(1) $$SINT=\rho \left(1-e^{-TR\left(\frac{1}{T_{1m}}+r_{1}C\right)}\right)e^{-TE\left(\frac{1}{T_{2m}}+r_{2}C\right)}$$

where ρ is the proton density, *TE* and *TR* denote the echo time and repetition time, *T*_1m_ and *T*_2m_ represent the longitudinal and transverse relaxation time of the matrix without the contrast agent, *r_1_* and *r_2_* are the longitudinal and transverse relaxivities of the contrast agent, respectively, and *C* is the concentration of the contrast agent. Then, the MRI signal enhancement efficiency (SEE) of a given contrast agent can be described by

(2) $$\begin{array}{ccc} SEE & = & \frac{\Delta SINT}{\rho }=\left(1-e^{-TR\left(\frac{1}{T_{1m}}+r_{1}C\right)}\right)e^{-TE\left(\frac{1}{T_{2m}}+r_{2}C\right)}\\ & & -\left(1-e^{-\frac{TR}{T_{1m}}}\right)e^{-\frac{TE}{T_{2m}}} \end{array}$$

where *∆SINT* is the signal enhancement induced by the contrast agent. Obviously, *SEE* is a function of the concentration of contrast agent under given imaging parameters and matrix. In addition, it is also sensitive to *r*_1_ and *r*_2_.

To show the concentration dependency of SEE, aqueous solutions of PUSIONPs with concentrations ranging from 0.01 to 10 mm were imaged by using the T1‐FSE sequence. The T1‐weighted images exhibited a brightening followed by an unexpected dimming trend when the iron concentration was above 3 mm, as shown in Figure S4A (Supporting Information). The experimentally extracted SEE values were then compared with the theoretical values predicted by Equation 2 in Figure S4B (Supporting Information), and a correlation coefficient as high as 0.997 was obtained. It is worth mentioning that *r*_1_ and *r*_2_ for calculating SEE were extracted by using PUSIONPs aqueous solutions with concentrations ranging from 0.05 to 0.5 mm, much lower than those for the SEE experiment. Therefore, the high correlation coefficient on the one hand indicated that the PUSIONPs remained dispersive rather than forming aggregates even at 10 mm, and on the other hand suggested that the dimming trend for the T1 signal was caused by a concentration effect rather than particle aggregation.

### 2.4 Biodistribution of PUSIONPs through SPECT

**Figure 3:**
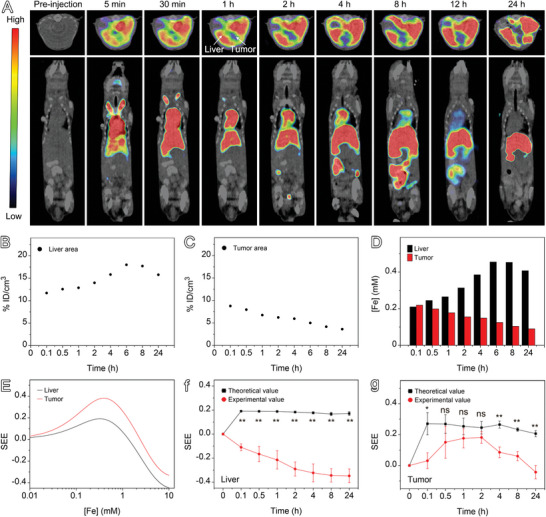
Biodistribution of PUSIONPs in liver and tumor. A–C) SPECT/CT images of HCC‐bearing mice acquired after intravenous injection of ^99m^Tc‐labeled PUSIONPs (A), together with γ‐signals of the liver (B) and tumor (C), determined at different time points post‐injection. D) Temporal concentrations of PUSIONPs in the liver and tumor. E) Theoretical SEE‐[Fe] curves obtained by assuming that PUSIONPs are evenly distributed in both the liver and tumor. F,G) Comparisons of theoretically predicted and experimentally determined SEE values of liver (F) and tumor (G) at different time points post‐injection. Data are presented as mean ± SD, *n* = 3. *p*‐values were calculated using a unpaired Student's *t*‐test. Statistical significance is indicated as follows: **p < 0.01, *p ≤ 0.05, ns = not significant (*p* > 0.05).

Based on the analysis provided above, it can be deduced that the extraordinarily high contrast for HCCs is probably caused by differences in the concentration of PUSIONPs in hepatic parenchyma and tumor tissues. To verify this hypothesis, dynamic mapping of PUSIONPs in HCC‐bearing mice was carried out through single photon emission computed tomography (SPECT) after PUSIONPs were radiolabeled with ^99m^Tc using a previously established LAGMERAL method^[^ ^25^ ^]^ The resulting radioactive probe exhibited excellent radiolabeling stability over 24 h incubation in PBS and Dulbecco's modified Eagle's medium (DMEM) containing 10% fetal bovine serum (FBS) (Figure S5, Supporting Information), well satisfying the prerequisites for quantitatively disclosing the concentration‐dependent relaxometric properties of PUSIONPs. The SPECT/CT images of HCC‐bearing nude mice receiving intravenously delivered ^99m^Tc‐labeled PUSIONPs (0.1 mmol Fe/kg body weight) are shown in **Figure** **3**A. The quantitative signals from the volume of interest expressed as a percentage of the injected dose per cubic centimeter (% ID/cm^3^) are given in Figure 3B,C. It can be seen that PUSIONPs are mainly distributed in blood and blood‐rich organs in the initial stage of the post‐injection period, and the signal of the liver is much higher than that of the tumorous site, which appears as a relatively low signal area in comparison with surrounding liver tissues. With the lapse of time, PUSIONPs are gradually cleared from the blood, and the liver signal gradually increases within the initial 6 h due to the uptake of PUSIONPs. Afterward, the signal is attenuated due to the excretion of PUSIONPs into the intestine, which is evidenced by the appearance of a signal in the abdominal cavity. In difference, the signal intensity of tumor all the way decreases against time.

Based on the biodistribution of PUSIONPs obtained through SPECT, the concentrations of nanoparticles in liver and tumor at different time points were determined. However, liver is a blood‐rich organ, and the interference of nanoparticles in the blood vessels must be excluded to obtain the temporal concentration of PUSIONPs in the hepatic parenchyma. Therefore, the radioactive signal of the heart region was first analyzed to obtain the blood circulation profile. As indicated in Figure S6 (Supporting Information), the blood half‐life of the PUSIONPs was calculated to be 2.1 h by using a two‐compartment model, which is much longer than those for clinical gadolinium‐based contrast agents (several minutes in small animals)^[^ ^26^ ^]^ and other iron oxide nanoparticles.^[^ ^3^, ^27^ ^]^ Based on the pharmacokinetic behavior and the calculation procedures given in the Supporting Information, the temporal concentrations of PUSIONPs in the hepatic parenchyma and the tumor site were obtained as shown in Figure 3D. By assuming that PUSIONPs are uniformly distributed in the region of interest, the SEE‐[Fe] curves were drawn according to Equation (2) by using the experimentally determined relaxation times and the scanning parameters in Table S1 (Supporting Information). Furthermore, based on the temporal concentration and the correlation between SEE and [Fe] in Figure 3D,E, the temporal SEE values of hepatic parenchyma and tumor, both calculated and experimentally determined, were obtained and are presented in Figure 3F,G. It is quite obvious that the theoretical SEE values of both hepatic parenchyma and tumor present quite different signal variations from the experimental SEE values obtained according to the results in Figure 2A. The remarkable inconsistency indicates that the hypothesis on the uniform distribution of PUSIONPs in the liver and tumor may not be true.

### 2.5 Microscopic Distribution of PUSIONPs at Tissue, Cellular, and Subcellular Levels

To reveal the microscopic distribution of PUSIONPs, the HCC‐bearing mice were sacrificed 4, 8, and 24 h post‐injection of PUSIONPs, and then the tissues of interest were harvested and subjected to Prussian blue staining to assess the microscopic distribution of PUSIONPs. As shown in Figure S7 (Supporting Information), the accumulation of PUSIONPs in the hepatic parenchyma was significantly heavier than that in the tumor, consistent with the SPECT results in Figure 3. Most importantly, the PUSIONPs were unevenly distributed at the microscopic scale.

It is well known that liver is a complex network of interrelated cells, mainly including hepatocytes (HCs), liver sinusoidal endothelial cells (LSECs), Kupffer cells (KCs), hepatic stellate cells (HSCs), and large granular lymphocytes (LGLs).^[^ ^28^ ^]^ The blood from both the hepatic artery and portal vein mixes together in hepatic sinusoids, which contain a variety of liver cells, including LSECs, KCs, and LGLs.^[^ ^28^ ^]^ After intravenous administration, PUSIONPs carried by the bloodstream will flow through the hepatic sinusoids and penetrate the fenestration into the Disse space due to their ultra‐small size, then interact with various kinds of liver cells. To further disclose the cellular level distribution, PUSIONPs were labeled by FITC, as schematically illustrated in Figure S8A (Supporting Information), to obtain a fluorescent probe denoted as PUSIONPs‐FITC. The labeling processes exhibited a negligible impact on the morphology of the nanoprobes according to the transmission electron microscopy (TEM) results in Figure S8B,C (Supporting Information). The conventional optical spectroscopy results in Figure S8D,E (Supporting Information) indicated that FITC was successfully conjugated to PUSIONPs. Further DLS measurement results in Figure S8F (Supporting Information) indicated that the as‐prepared PUSIONPs‐FITC displayed a hydrodynamic size profile very similar to that of the mother nanoparticles. Moreover, as demonstrated in Figure S8G (Supporting Information), PUSIONPs‐FITC exhibited excellent labeling stability in water, simulated body fluid (SBF), and phagolysosomal simulant fluid (PSF), providing a reliable opportunity for tracing PUSIONPs through FITC.

**Figure 4:**
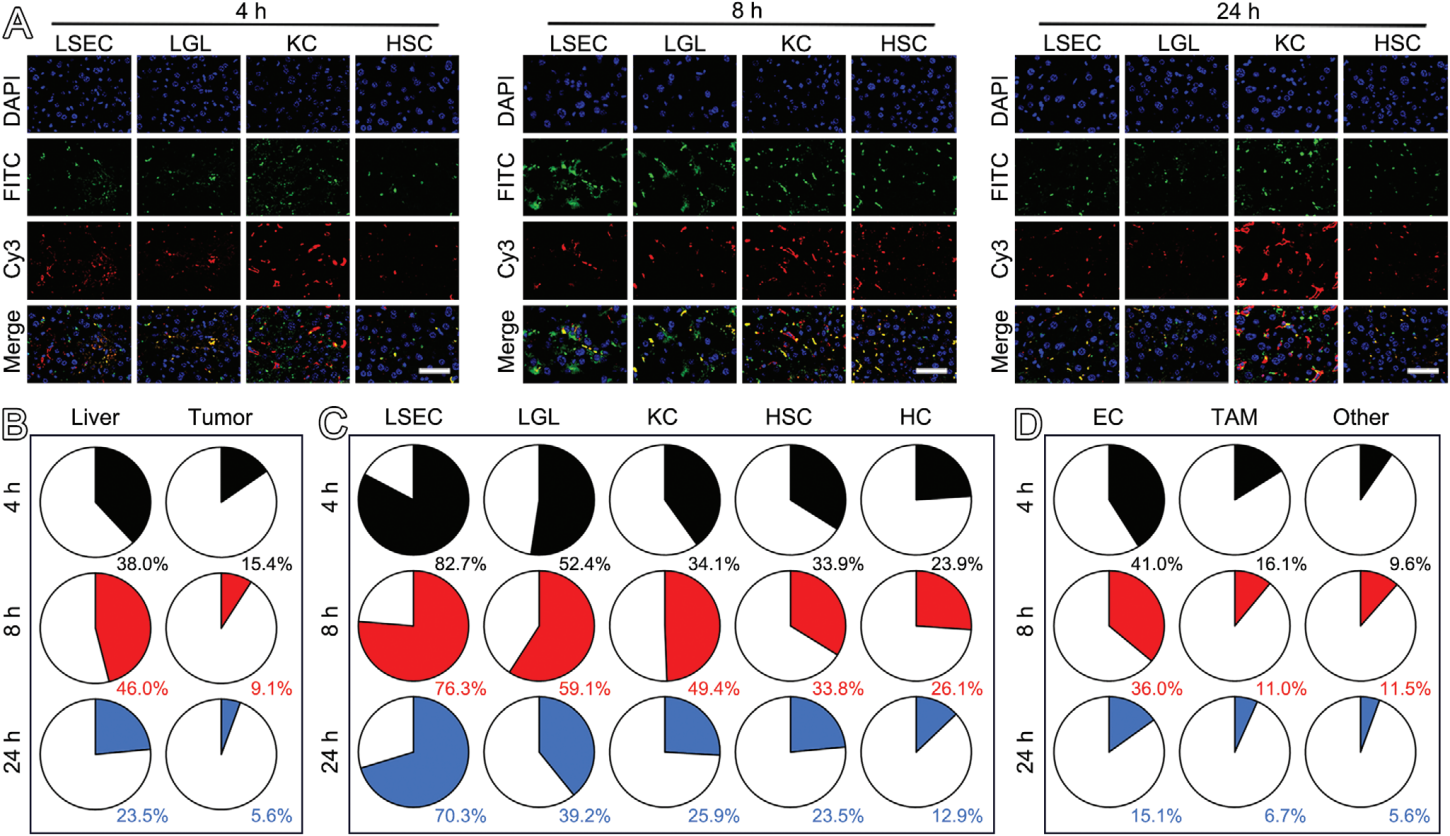
Distribution of PUSIONPs at tissue and cellular level. A) Immunofluorescence images of liver tissues extracted at different time points post intravenous injection of PUSIONPs‐FITC (the scale bar corresponds to 10 µm). Each condition was replicated in three different mice (biological replicates), *n* = 3. B) Percentage of PUSIONPs‐positive cells in the liver and tumor at different time points post‐injection. Data are presented as mean, *n* = 3. C,D) Temporal variations in PUSIONPs‐positive cell ratios for each kind of liver cell (C) and tumor cell (D). Data are presented as mean, *n* = 3.

To monitor the cellular level distribution of PUSIONPs, the HCC‐bearing mice were sacrificed 4, 8, and 24 h post‐injection of PUSIONPs‐FITC to extract the organs and tissues of interest. Then, they were subjected to immunofluorescence analysis after being cut into 5 µm‐thick slices, followed by staining with IYVE1, CD49b, F4/80, and GFAP antibodies labeled with Cy3 for identifying LSECs, LGLs, KCs, and HSCs, respectively. It can be seen from the confocal microscopy images in **Figure** **4**A that all liver cells are simultaneously stained green by FITC on PUSIONPs and red by Cy3 on antibody, apart from blue by DAPI to show the cell nucleus, confirming that PUSIONPs are internalized into these types of liver non‐parenchymal cells, although by different uptake levels. To show the colocalization level of green and red fluorescence from different liver cells, Pearson's correlation coefficient (PCC) was calculated using ImageJ software. The results tabulated in Table S2 (Supporting Information) revealed non‐monotonic variations in PCCs for KCs and HSCs and monotonic variations in PCCs for LSECs and LGLs. The inconsistent trends suggest that the cellular uptake behaviors of PUSIONPs by various liver cells are also different. Moreover, in comparison with the liver, the tumor area presented much weaker green fluorescence, as shown in Figure S9 (Supporting Information), indicating that the uptake level of PUSIONPs in the tumor was remarkably lower, well in accordance with the SPECT and Prussian blue staining results given in Figure 3 and Figure S7 (Supporting Information), respectively. In addition, the confocal microscopy images in Figure S9 (Supporting Information) and PCC results in Table S2 (Supporting Information) revealed that the green fluorescence is notably co‐localized with the red fluorescence of endothelial cells (ECs) and tumor‐associated macrophages (TAMs) that also overexpress IYVE1 and F4/80, respectively, indicating that PUSIONPs are largely uptaken by ECs and TAMs after reaching the tumor site.

To quantify the cellular uptake of PUSIONPs, HCC‐bearing mice were sacrificed 4, 8, and 24 h after intravenous injection of FITC‐labeled PUSIONPs. After perfusion, the liver and tumor harvested were subjected to digestion to obtain single‐cell suspensions for further flow cytometry analysis. Figure 4B and Figure S10 (Supporting Information) show the percentage of PUSIONPs‐positive cells in the liver and tumor at different time points post‐injection. As expected, the proportion of PUSIONPs‐positive cells in the liver was significantly higher than that in the tumor, irrespective of the post‐injection time. In addition, the signal of the liver first increased and then decreased, while the tumor exhibited a continual decline, consistent with the organ‐level distributions of PUSIONPs obtained through SPECT (Figure 3). Furthermore, nearly half of the cells (≈46%) in the liver presented green fluorescence 8 h post‐injection, indicating that PUSIONPs are prone to be uptaken by liver cells. Actually, PUSIONPs delivered through the hepatic artery and portal vein accumulate in hepatic sinusoids, where some particles are sequestered by LSECs, LGLs, and KCs, while the rest will enter Disse space to interact with HCs and HSCs. To disclose the internalization efficiency of PUSIONPs into different liver cells, the isolated liver cells were stained with anti‐CD146‐PE, anti‐CD49b‐PE, anti‐F4/80‐BV421, and anti‐GFAP‐AF647 antibodies, respectively, to detect LSECs (Figure S11, Supporting Information), LGLs (Figure S12, Supporting Information), KCs (Figure S13, Supporting Information), and HSCs (Figure S14, Supporting Information). Due to the lack of specific markers, HCs that were not stained by the abovementioned antibodies were categorized as the “other” population (Figure S15, Supporting Information). Figure 4C summarizes the temporal ratios of each kind of PUSIONPs‐positive liver cell. Obviously, all types of liver cells exhibit a certain uptake capacity for PUSIONPs. Among them, LSECs show the highest uptake capacity, which is inagreement with previous studies supposing that ultra‐small iron oxide nanoparticles had a similar behavior to their larger counterparts, which are mainly phagocytized by KCs after entering the liver.^[^ ^29^ ^]^ In addition, there is also a discrepancy between the current results and those reported by Chan and coworkers on PEG 5K‐modified CdSeS/ZnS quantum dots with a core size of ≈4.7 nm and a hydrodynamic size of ≈14 nm.^[^ ^30^ ^]^ In the latter study, KCs exhibited the highest uptake capacity, although LSECs also presented a significant accumulation. In addition, quantum dots were not detected in HCs. The differences between PUSIONPs and PEGylated CdSeS/ZnS quantum dots suggest that the uptake of liver cells is very sensitive to the length of the PEG ligand, which was also observed through careful studies on the chain length of PEG ligands of iron oxide particles.^[^ ^31^ ^]^ Interestingly, a similar phenomenon was observed in tumor where ECs presented a higher uptake capacity than TAMs, as shown in Figure 4D, Figures S16 and S17 (Supporting Information), further demonstrating that PUSIONPs are more prone to be uptaken by endothelial cells rather than macrophages.

**Figure 5:**
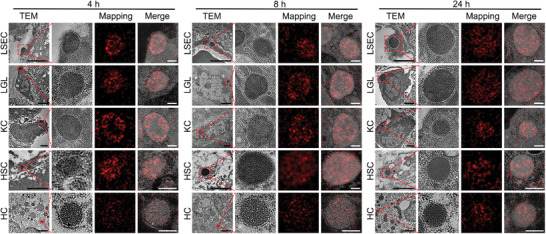
Distribution of PUSIONPs at subcellular level. TEM images (first and second rows) and iron mapping of liver tissues through STEM (third row) taken at different time points post‐injection of PUSIONPs to show the distribution of PUSIONPs at the cellular level. The black scale bar corresponds to 2 µm, and the white scale bar corresponds to 200 nm.

To further disclose the distribution of PUSIONPs at the subcellular level, TEM and scanning TEM (STEM) were employed to examine the liver samples obtained at different time points post‐injection. As shown in **Figure** **5**, through the relative positions and morphologies, different types of liver cells can be unambiguously distinguished. In combination with the elemental mapping of iron and elemental analysis (Table S3, Supporting Information), it was found that PUSIONPs were distributed in all the examined cells. Regardless of the cell type, most PUSIONPs were notably sequestered into endosomes, while the cytosol and other intracellular compartments (e.g., mitochondria, nucleus, and reticulum) remained almost unoccupied. In a previous study, IONPs were found to mainly locate in the lysosomes of liver cells such as KCs.^[^ ^29b^ ^]^ It was also believed that once within lysosomes, IONPs would form aggregates to enhance T2 imaging contrast.^[^ ^2^, ^19^, ^32^ ^]^ However, careful observation of the TEM images in Figure 5 reveals that PUSIONPs are more likely dispersive rather than forming clusters in endosomes. In fact, owing to the extremely strong binding affinity of DP‐PEG to the particle surface, PUSIONPs displayed a nearly unchanged hydrodynamic size after being incubated in PSF (pH 4.5) for 10 d (Figure S18, Supporting Information), suggesting that PUSIONPs can withstand the endosome pH to remain dispersive. Therefore, it was hypothesized that the strong hypointense signal from the hepatic parenchyma, as shown in Figure 2, was caused by the concentration effect of the dispersive IONPs in endosomes rather than the aggregation effect of IONPs, as stated in previous studies.^[^ ^12^ ^]^

### 2.6 Concentration‐Dependent MRI Contrast Effects of PUSIONPs‐In‐Liposomes

To verify the above speculation, liposomes containing different concentrations of PUSIONPs were prepared to simulate the cell endosomes hosting the nanoparticles. The iron concentration inside the liposomes was denoted as [Fe]_liposome_ to distinguish it from the average iron concentration in solution, i.e., [Fe]. Figure S19 (Supporting Information) shows a representative TEM image and the size distribution histogram of the as‐prepared PUSIONPs‐containing liposomes. It can be seen that the sizes of PUSIONPs‐containing liposomes ranged from 50 to 500 nm, consistent with those for endosomes (25–800 nm).^[^ ^33^ ^]^ In addition, PUSIONPs were well dispersed inside the liposomes (Figure S19A, Supporting Information). Moreover, increasing the concentration of PUSIONPs did not lead to unwanted aggregation, as evidenced by the small variation in the hydrodynamic size of liposomes (Figure S19C, Supporting Information). Altogether, the as‐prepared liposomal structure can be taken as a satisfactory model for mimicking cellular endosomes containing different concentrations of PUSIONPs inside.

**Figure 6:**
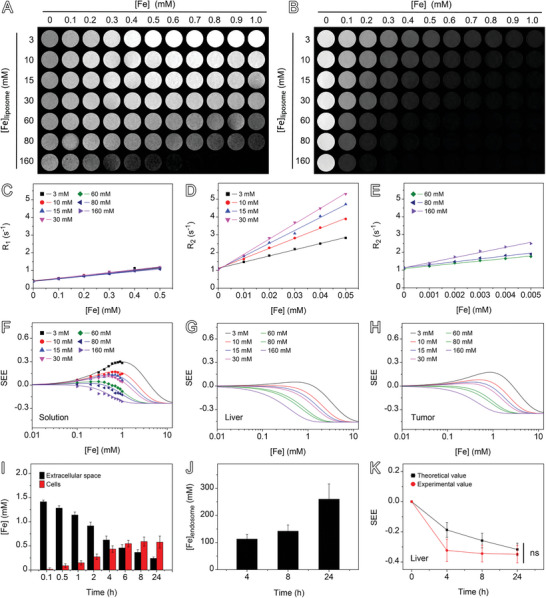
Experimentally determined and theoretically derived SEE values. A,B) T1‐weighted (A) and T2‐weighted (B) MR images of aqueous solutions of liposomes containing PUSIONPs with different [Fe]_liposome_ and [Fe]. C–E) Experimental data together with the corresponding theoretical fittings (solid lines) for extracting *r*_1_ (C) and *r*_2_ (D‐E) of PUSIONPs‐in‐liposome. F) Comparison of experimentally determined SEE values with the theoretical SEE‐[Fe] curves of liposomes containing different concentrations of PUSIONPs, obtained with Equation 2 by employing the relaxivity values extracted from the data in panels (C–E). g,h) Theoretical SEE‐[Fe] curves for liver (G) and tumor (H) at given [Fe]_endosome_ ranging from 3 to 160 mm. I) Temporal iron concentration in liver cells and extracellular spaces after intravenous injection of PUSIONPs. Data are presented as mean ± SD, *n* = 3. J) Temporal [Fe]_endosome_ in liver cells. Data are presented as mean ± SD, *n* = 3. K) Experimentally determined and theoretically derived SEE values of liver for different time points post‐injection of PUSIONPs. Data are presented as mean ± SD, *n* = 3, *p*‐value was calculated using unpaired Student's t test, ns = not significant (*p* > 0.05).

**Figure** **6**A,B shows T1‐ and T2‐weighted images of a series of liposome solutions containing different concentrations of PUSIONPs. It can be seen that the liposomes showed a significant T1 enhancement effect when the [Fe]_liposome_ was 3 mM. However, the T1 enhancement effect is gradually suppressed and even got vanished with the increase of [Fe]_liposome_. In the meantime, the T2 enhancement effect is remarkably improved. These phenomena are superficially similar to the T1‐quenching and T2‐enchancing effects caused by the aggregation of magnetic nanoparticles.^[^ ^2^, ^12^ ^]^ To quantitatively evaluate the relaxometric performances of liposomes with different concentrations of Fe, *r*_1_ and *r*_2_ of the liposomes were measured by linear aggressive fitting of concentration‐dependent R_1_ and R_2_, as shown in Figure 6C–E and Table S4 (Supporting Information). The fitting results revealed that *r*_1_ decreased from 7.9 mm ^−1^ s^−1^ for PUSIONPs to ≈1.5 mm ^−1^ s^−1^ for PUSIONPs‐in‐liposome, irrespective of [Fe]_liposome_, while *r*_2_ increased significantly from 31.4 to 288.0 mm ^−1^ s^−1^ when [Fe]_liposome_ was raised from 3 to 160 mM. Based on these *r*_1_ and *r*_2_ values, the [Fe]‐dependent SEE of liposomes containing different concentrations of PUSIONPs was calculated and compared with the experimentally determined SEE results according to the results in Figure 6A,B. In fact, owing to the remarkably strong [Fe]_liposome_ dependency, the concentration of Fe had to be largely decreased for measuring R_2_. In other words, the [Fe] for extracting *r*_2_ is much lower than those used for determining SEE. Therefore, the excellent consistency between the experimental and theoretical results in Figure 6F suggests that the MRI contrast enhancement effects of PUSIONPs‐in‐liposomes can well be predicted if they are taken as a new type of contrast agent with greatly increased *r*_2_. Thus, by using the *r*_1_ and *r*_2_ values of PUSIONPs‐in‐liposomes with different [Fe]_liposome_, the SEE values were calculated and plotted against [Fe] for predicting the SEE values of the liver and tumor at a given [Fe]_liposome_. The results in Figure 6G,H reveal that the hypointense signal is easily generated with a low concentration threshold for PUSIONPs in the endosomes of liver cells, while this concentration threshold is much higher for PUSIONPs in tumor. Therefore, it can be deduced that the difference in the local concentrations of iron in the tumor and liver may explain the extraordinarily high liver‐to‐tumor contrast for HCCs.

It is known that the extracellular spaces in hepatic parenchyma are directly connected to the blood system. Therefore, it can be assumed that the concentration of PUSIONPs in these spaces is the same as that in the blood. Based on this assumption, in combination with the temporal PUSIONPs concentrations in the hepatic parenchyma (Figure 3D) and the blood residence profile (Figure S6, Supporting Information), the average iron concentration in liver cells and extracellular spaces were calculated and presented in Figure 6I, with the detailed derivation procedures being described in SI. As PUSIONPs were mainly observed in endosomes of hepatic cells and four other non‐parenchyma cells (Figure 5), the concentration of PUSIONPs in the endosomes, i.e., [Fe]_endosome_ was derived and shown in Figure 6J, based on the PUSIONPs‐positive ratio of cells (Figure 4C), the volume ratio of endosomes to liver cells, and the volume ratio of cells to liver (Table S5, Supporting Information). Then, combining the results given in Figure 6G, the temporal SEE values of the liver were calculated as displayed in Figure 6K. It can be seen that the theoretical values match perfectly with the experimental values, confirming that the concentration effect of PUSIONPs in endosomes rather than their aggregation leads to the hypointense signal in the liver.

By using similar procedures mentioned above, the concentrations of PUSIONPs in both the blood vessels and matrix of the tumor were obtained, as shown in Figure S20A (Supporting Information). The concentration of PUSIONPs in the tumor matrix is much lower than that in the liver, e.g., only one fifth of that for liver 8 h post‐injection. Owing to the heterogeneity of tumor, the volume fraction of tumor cells and local concentration of PUSIONPs in the endosomes of tumor cells are difficult to estimate. Nevertheless, taking the differences between [Fe]_liver_ and [Fe]_endosome_ in hepatic parenchyma (Figure 6I,J) into consideration, it is reasonable to infer that [Fe]_endosome_ is at least two orders of magnitude higher than the average [Fe] in the tumor matrix. By this assumption, the SEE values of the tumor site were estimated. As shown in Figure S20B (Supporting Information), the theoretical values were very close to the experimental results. In contrast, as aforementioned, if PUSIONPs are evenly distributed in the tumor region, the theoretical values of SEE are far from the experimental values (Figure 3G).

### 2.7 Mechanism of Exceptionally High Tumor‐To‐Liver Contrast for HCC Diagnosis

**Figure 7:**
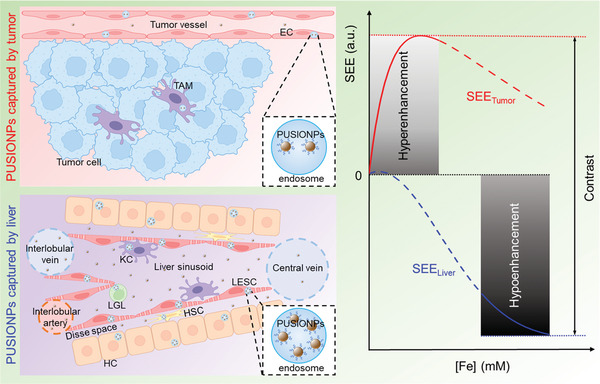
Schematic drawing of the mechanism for HCC Diagnosis. Schematic drawing showing the mechanism underlying the extremely high tumor‐to‐liver contrast in PUSIONPs‐enhanced MRI of HCCs.

Therefore, putting the theoretical results and experimental data together, we propose the following mechanism for explaining the extremely high tumor‐to‐liver contrast in **Figure** **7**. After intravenous administration, PUSIONPs are rapidly and extensively internalized by various liver cells, including LSECs, LGLs, KCs, HSCs, and HCs, and sequestered in endosomes, which leads to remarkably increased *r*_2_ values and the consequent hypointense MRI signal of hepatic parenchyma on T1‐weighted imaging. In contrast to the liver, although PUSIONPs are also enriched in the endosomes of tumor cells, due to the much lower uptake efficiency of tumor cells, the [Fe]_endosome_ in tumor falls in the range where hyperintense signals are dominant on T1‐weighted imaging. In fact, it is easily understood that the local concentration of PUSIONPs in liver is much higher than that in tumor. 1) Liver is a blood rich organ. 2) There are many different types of cells in the liver involved in the uptake of nanoparticles. 3) The velocity for nanoparticles to pass through the sinusoid is 3 orders of magnitude lower than that for particles to traverse arteries and veins.^[^ ^30^ ^]^ 4) The intravenously delivered nanoparticles have the opportunity to directly contact liver cells, while the nanoparticles in blood need to cross the wall of blood vessels prior to being uptaken by tumor cells.^[^ ^34^ ^]^ These differences make the local concentration of PUSIONPs simultaneously fall in the hyperintense signal region for the tumor and hypointense signal region for the liver on T1‐weighted imaging, perfectly maximizing the contrast of HCCs.

In fact, many previous studies have significantly contributed to the development of MRI contrast agents based on ultra‐small magnetic nanoparticles,^[^ ^16^, ^17^, ^18^ ^]^ while these previous studies were more focusing on the optimization of relaxivities through the structural parameters of the nanometer‐sized contrast agents. In difference, the current studies suggest that the dispersion status of the PUSIONPs is also an important parameter, providing an outstanding performance for imaging HCCs, e.g., to achieve simultaneous hyperintense signals in HCC tumors and hypointense signals in the liver parenchyma. This dual‐contrast capability significantly improves the tumor‐to‐liver contrast ratio, enhancing the sensitivity and accuracy of HCC diagnosis. To the best of our knowledge, this dual‐contrast mechanism has not been reported in previous studies.

### 2.8 Dose‐Dependent Imaging Contrast and Detection of Tiny HCCs Using PUSIONPs

**Figure 8:**
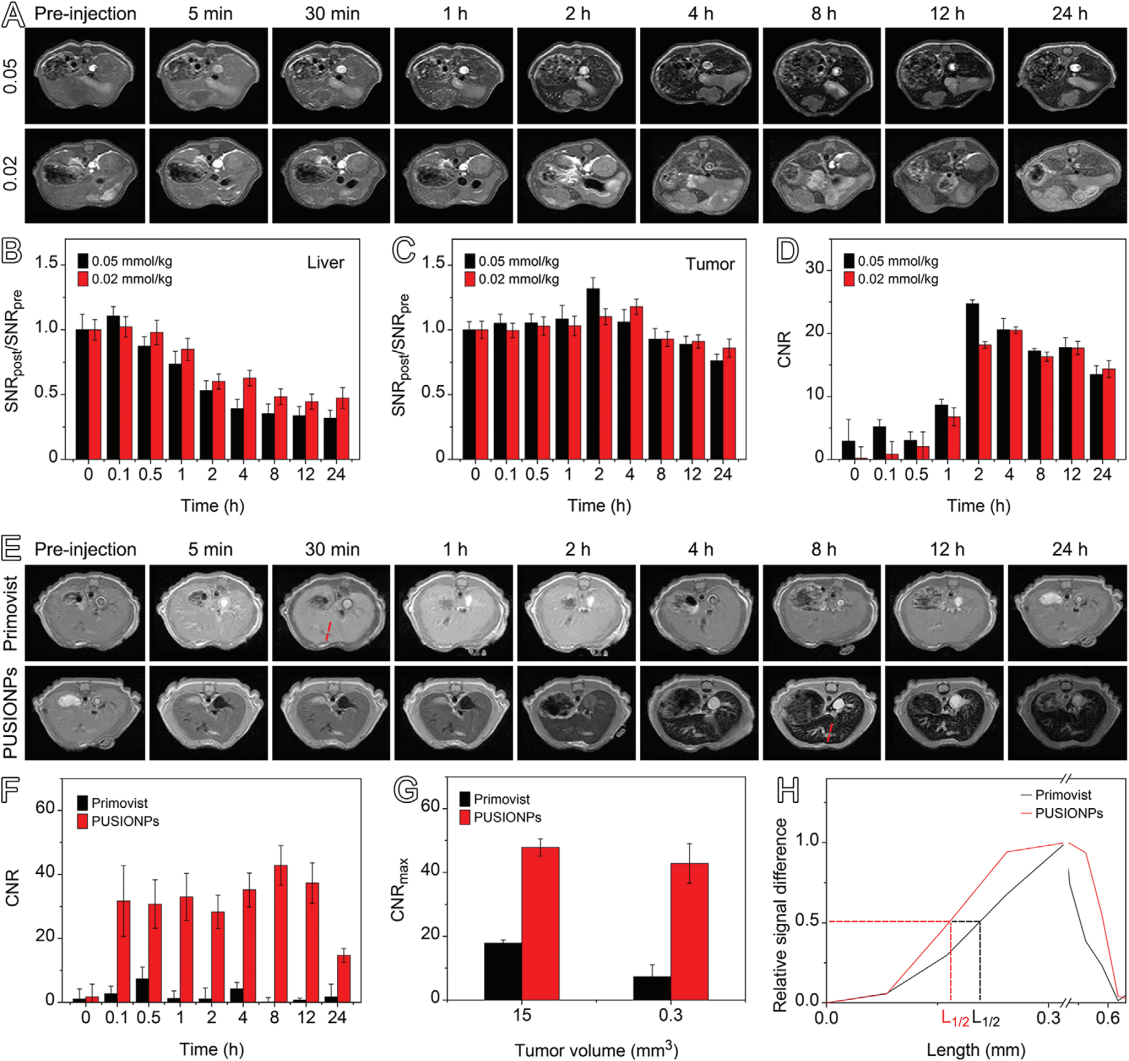
Dose‐dependent MR imaging of PUSIONPs. A) T1‐weighted MR images of HCC‐bearing mice acquired before and at different time points after intravenously injecting PUSIONPs at doses of 0.05 and 0.02 mmol Fe per kg body weight, respectively. B,C) Relative SNR values of the hepatic parenchyma (B) and tumor (C) at different time points post‐injection. D) CNR values of the tumor site after injecting PUSIONPs at different doses. E) T1‐weighted MR images of a mouse bearing HCC of 0.3 mm^3^ enhanced by Primovist and PUSIONPs, respectively, with a dose of 0.1 mmol Gd/Fe per kg body weight. F) Temporal CNR values of the tiny tumor. G) The maximum CNR values for HCCs of different sizes obtained with Primovist and PUSIONPs. H) Profiles of the relative signal drawn along the red lines in the MRI images in panel (E). For insets (B–D) and (F,G), data are presented as mean ± SD, *n* = 1. The mean stands for signal intensity in the ROI and SD stands for the standard deviation analyzed from the ROI of MR images.

The above mechanism discloses that the contrast enhancement is strongly concentration‐dependent when PUSIONPs are confined in endosomes, it is therefore reasonable to deduce that the injected dose of PUSIONPs may become a crucial parameter for the imaging contrast of PUSIONPs‐enhanced MRI of HCCs. To evaluate the dose effect, T1‐weighted MRI studies were further performed on HCC‐bearing mice before and after intravenous administration of PUSIONPs at doses of 0.05 and 0.02 mmol Fe/kg body weight, respectively. Compared to the results given in Figure 2A (0.1 mmol Fe/kg body weight), the tumor region exhibits a similar signal trend, as shown in **Figure** **8**A. However, different from the high‐dose experiments in which hypoenhancement effects appear in the hepatic parenchyma immediately after the injection, slow hyperenhancement appears followed by hypoenhancement in the low‐dose groups. In addition, the time for the signal intensity of tumor site to reach its maximum is slightly delayed with the decrease of dose level, e.g., 0.5, 2, and 4 h for 0.1, 0.05, and 0.02 mmol Fe/kg body weight, respectively. This is because the lower the injection dose is, the longer period of time the tumor needs to uptake sufficient PUSIONPs to reach the signal maximum. It is worth mentioning that decreasing the injection doses is unfavorable for CNR if comparing the results in Figure 8D with those in Figure 2C, the CNR values obtained with the lowest dose level of PUSIONPs (0.02 mmol Fe/kg body weight) remain higher than those obtained with Primovist (0.1 mmol Fe/kg body weight), well manifesting the advantage of PUSIONPs in HCC diagnoses.

To show the potential of PUSIONPs for diagnosing tiny HCCs, the contrast enhancement effects of PUSIONPs were systematically evaluated on HCCs of smaller size, e.g., 0.3 mm^3^, with Primovist serving as a control. In detail, the HCC‐bearing mice were injected successively with Primovist and PUSIONPs at an interval of 24 h. The T1‐weighted images were captured and shown in Figure 8E. As given in Figure S21 (Supporting Information), the liver and tumor exhibited different signal‐varying trends after the administration of PUSIONPs, consistent with the results on HCC of 15 mm^3^ (Figure S3, Supporting Information). Similar to the abovementioned results on HCC of 15 mm^3^ (Figure 2A), PUSIONPs provide significantly higher imaging contrast than Primovist for HCC of 0.3 mm^3^. In addition, the quantitative CNR and tumor boundary clarity, as presented in Figure 8F–H and Table S6 (Supporting Information), further demonstrate that PUSIONPs are capable of providing higher contrast and better boundary clarity than Primovist irrespective of the tumor size. Consistent with previous studies,^[^ ^6^, ^7^, ^9^, ^22^ ^]^ the CNR value achieved with Primovist sharply decreased with decreasing tumor size. In contrast, the CNR value obtained with PUSIONPs is not only higher but also remarkably stable with decreasing tumor size, which makes PUSIONPs particularly suitable for the diagnosis of early HCCs.

### 2.9 Theoretical Prediction of HCC Diagnosis with PUSIONPs in Clinical Settings

**Figure 9:**
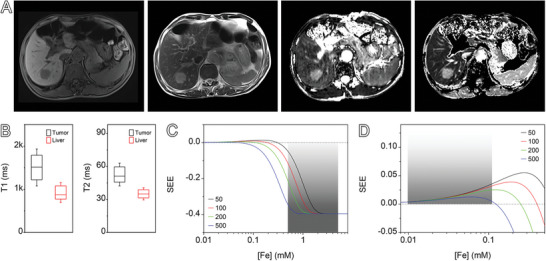
Theoretical SEE prediction for HCC patients. A) Representative T1‐weighted, T2‐weighted, T1 mapping, and T2 mapping images of an HCC patient acquired by MRI scans without contrast agent. B) Relaxation time data for the liver and tumor regions from 15 HCC patients. Data are presented as mean ± SD, *n* = 15. C,D) Theoretical curves depicting SEE as a function of average [Fe] for the liver (C) and tumor (D) at specified enrichment levels, with [Fe]_endosome_ exceeding the average [Fe] in the liver or tumor matrix by 50–500 times.

The above pre‐clinical mouse model studies have demonstrated the remarkable potential of PUSIONPs for more precise and sensitive diagnosis of HCCs. Clinical imaging data based on PUSIONPs from actual patients remain unavailable yet. Therefore, prior to future clinical trials, it would be necessary to predict the imaging effects of PUSIONPs in HCC patients based on the conceptual framework proposed in the current study. As discussed above, apart from imaging parameters, the liver‐to‐tumor contrast also hinges on the relaxation times of both anatomical regions and the enrichment degree of PUSIONPs therein, which may be different between mice and humans. Therefore, MRI scans were conducted on 15 HCC patients (**Figure** **9**A), to determine the pre‐contrast longitudinal and transverse relaxation times of the liver and tumor, as statistically depicted in Figure 9B. Considering the endosomal enrichment of PUSIONPs in various types of cells, we can reasonably posit, based on animal‐level data, that the [Fe]_endosome_ surpasses the average [Fe] in the liver or tumor matrix by 50–500 times. Under this assumption, the SEE values of the liver and tumor regions were calculated and plotted against [Fe] in Figure 9C. Given that the hydrodynamic size of PUSIONPs exceeds 10 nm, the limited renal excretion makes liver uptake dominate the biodistribution. As estimated in the Supporting Information, an injection dose of 0.1 mmol kg^−1^ will give rise to an average liver [Fe] ranging from 0.5 to 4 mm. Whereas, the enhanced permeability and retention (EPR) effect of solid tumors only leads to comparatively modest tumor accumulation. As described in the Supporting Information, the average [Fe] in tumors is estimated to be an order of magnitude lower than that in normal liver tissue, i.e., 0.01 to 0.1 mm. Thus, within these estimated ranges of average [Fe], the SEE values calculated based on the imaging results of HCC patients reflect that the tumor region will exhibit a hyperintense signal, while the liver will present a hypointense signal, as illustrated in Figure 9C. This persistent manifestation of the reversed contrast‐enhancing effects establishes a theoretical basis for the high‐sensitivity diagnosis of HCC patients with PUSIONPs as magnetic resonance contrast agents.

## 3 Discussion

In summary, the highly sensitive MRI diagnosis of tiny HCCs particularly those of sub‐millimeters has been successfully achieved through simultaneous hyperenhancement of tumor and hypoenhancement of hepatic parenchyma with PEGylated ultrasmall iron oxide nanoparticles as contrast agents. In‐depth studies reveal that PUSIONPs exhibit remarkable concentration‐dependent transverse relaxivity once they are trapped in cell endosomes. Consequently, PUSIONPs captured by liver parenchyma easily step into the hypoenhancement region, as the majority of liver cells are involved in the endocytosis of PUSIONPs, while the nano‐contrast agent in tumor falls in the hyperenhancement region owing to the much lower local concentration of nanoparticles in the endosomes of cancer cells. In addition, in comparison to Primovist, PUSIONPs exhibit much higher CNR and superior capability for delineating tumor boundaries, which enables the visualization of HCCs as small as 0.3 mm^3^ with much lower contrast agent doses. Most importantly, the integration of HCC patient‐derived imaging information and theoretical calculations supports the expectation of remarkably improved MR imaging contrast through the reversed contrast enhancement effects on HCC and its surrounding hepatic tissues. We thus believe that the current finding on the unique local concentration‐dependent transverse relaxivity makes PUSIONPs confined in cell endosomes particularly suitable for the highly sensitive diagnosis of HCCs, which lays a solid cornerstone for future clinical translation.

## 4 Experimental Section

### Chemicals

Ferric acetylacetonate (Fe(acac)_3_), purchased from Sigma‒Aldrich, was used after two recrystallizations. Other chemicals and reagents were used as received without any further purification. Oleic acid, oleylamine, 2‐aminoethanethiol, and tris(2‐carboxyethyl)phosphine (TCEP) were purchased from Sigma‒Aldrich. Stannous chloride (SnCl_2_) was purchased from Alfa Aesar. Other analytical grade chemical reagents, including isopropanol, acetone, ethanol, cyclohexane, and tetrahydrofuran (THF), were purchased from Sinopharm Chemical Reagent Co., Ltd, China. PEG derivatives, including DP‐PEG and DP‐PEG‐mal, were provided by Suzhou Xinying Bio‐Medical Technology Co. Ltd. Na^99m^TcO_4_ was purchased from Shanghai GMS Pharmaceutical Co., Ltd. The mouse liver cancer cell line Hepa1‐6 was obtained from Procell Co. Ltd. Dulbecco's modified Eagle medium (DMEM) (HyClone), Roswell Park Memorial Institute (RPMI)−1640 medium (HyClone), FBS (HyClone), penicillin and streptomycin were purchased from Beijing Biodee Biotechnology Co. Ltd. Fluorescein isothiocyanate (FITC) was purchased from Sinopharm. Milli‐Q water (>18 MΩ·cm) was used in the experiments.

### Characterization

TEM and scanning TEM images were captured using a Talos F200S G2 transmission electron microscope operating at an acceleration voltage of 200 kV. The hydrodynamic size and zeta potential were measured at 25 °C with a Malvern Zeta sizer Nano ZS90 equipped with a solid‐state He‐Ne laser (λ = 633 nm). The iron concentration was determined by the 1,10‐phenanthroline spectrophotometric method after the resulting nanoparticles were digested by HCl. In all experiments, the concentration of Fe_3_O_4_ NPs was presented by the concentration of iron. The relaxivity measurements were carried out on a 3 T preclinical MRI instrument (MRS300, MR Solutions). UV‒vis absorption spectra were recorded on a Shimadzu UV‒vis spectrophotometer UV‐3600 using quartz cuvettes with an optical path of 1 cm. The fluorescence spectra were recorded on an Edinburgh FLS 980 fluorescence spectrophotometer. Representative photomicrographs were taken by an Olympus FV 1200 laser scanning confocal microscope.

### Synthesis of Hydrophobic Ultra‐Small IONPs

Hydrophobic ultra‐small IONPs of 3.7 nm were prepared through flow synthesis based on thermal decomposition. Specifically, Fe(acac)_3_ (20 mmol), oleic acid (120 mmol), and oleylamine (120 mmol) were dissolved in 0.9 L of isopropanol. The solution was flushed with nitrogen for 10 min, followed by being pumped into the stainless‐steel tubular reactor by a high‐pressure pump with a flow rate of 30 mL min^−1^ under 5.2 MPa at 270 °C. After pyrolysis reactions, the resultant nanoparticles were precipitated and collected. By redispersion in cyclohexane and precipitation with acetone for three cycles, the ultra‐small IONPs were collected and dispersed in cyclohexane for further experiments.

### Ligand Exchange

Regarding the preparation of PEGylated ultra‐small IONPs (PUSIONPs), 500 mg of DP‐PEG was dissolved in 10 mL of THF containing 50 mg of hydrophobic ultra‐small IONPs. Then, the reaction mixture was heated to 40 °C and kept at this temperature for 48 h under stirring. After that, the nanoparticles were precipitated by cyclohexane, collected by centrifugation, washed with cyclohexane 3 times, and then dried under vacuum. After being dissolved in water, the resulting PUSIONPs aqueous solutions were subjected to ultrafiltration for three cycles using a 30 kDa MWCO centrifugal filter (Millipore YM‐30) to remove the excess PEG ligand. The PUSIONPs with surface maleimide residues were obtained by the same procedures as mentioned above by using DP‐PEG‐mal instead of DP‐PEG to replace the oleate ligand.

### Relaxivity Measurement

A series of solutions containing different concentrations of Magnevist, Primovist, or PUSIONPs were prepared for measuring the relaxivities. To measure the longitudinal relaxation time T1, an inversion recovery fast low‐angle shoot (IR FLASH) sequence was applied, and the T1 values were determined by analyzing the MRI images obtained with varied TR values (74–6170 ms) but a fixed TE value (6 ms). Regarding the transverse relaxation time T2, a multi‐echo multi‐slice (MEMS) sequence was applied, and the T2 values were obtained with varied TE values (15–450 ms) but a fixed TR value (1400 ms). *r*_1_ and *r*_2_ were then obtained through linear aggressive fitting of the correlations between longitudinal relaxation and iron concentration and transverse relaxation and iron concentration, respectively.

### ^99m^Tc Labeling of PUSIONPs

A 200 µL aqueous solution of Na^99m^TcO_4_ in saline with radioactivity of 4 mCi was first reduced by 20 µL of SnCl_2_ (1 mg mL^−1^ in 0.1 m HCl) at room temperature for 5 min. Then, an aqueous solution of PUSIONPs containing 0.2 mg Fe was introduced. The pH value of the reaction mixture was ≈3. After gently shaking for 30 min at room temperature, ^99m^Tc‐labeled PUSIONPs were obtained and purified by ultrafiltration using Millipore YM‐30 according to the standard protocol.

### Radiolabeling Stability Assessment

To evaluate the radiolabeling stability, the purified radiolabeled PUSIONPs were incubated in PBS or 10% FBS. Aliquots were extracted at different time points to monitor the radiochemical purities. Typically, 10 µL of the radiolabeled products were first mixed with 2 mL of Milli‐Q water and then concentrated to <100 µL through ultracentrifugation using a 30 kDa MWCO filter. The radioactivity of the residue remaining in the filter and the filtrate were measured by a gamma counter. The radiochemical purity was determined by comparing the activity retained in the filter with the total activity.

### Establishment of Orthotopic HCC Model

Hepa1‐6 cells were cultured in medium supplemented with 10% (v/v) FBS, 1 mm sodium pyruvate, and 1% (v/v) penicillin/streptomycin at 37 °C in an atmosphere of 5% CO_2_/95% air. BALB/c nude mice were anesthetized with chloral hydrate (4%) at a dose of 10 µL g^−1^. The orthotopic HCC tumor model was established by injection of Hepa1‐6 cells (≈5 × 10^5^) into the liver via laparotomy. The tumors were allowed to grow for 7–14 days to reach a volume of ≈0.3 and 15 mm^3^ for further experiments. All animal experiments reported herein were performed in accordance with the Guidelines for Care and Use of Laboratory Animals of Soochow University and approved by the Animal Ethics Committee of Soochow University. The assigned approval number is 202104A0358.

### In Vivo MR Imaging of HCC Tumor

The animals were anesthetized by a continuous supply of 1.5% isoflurane in oxygen. T1‐weighted imaging of animals was performed before and after the intravenous administration of Primovist or PUSIONPs at 5 min, 30 min, 1 h, 2 h, 4 h, 8 h, 12 h, and 24 h post‐injection by using the T1‐weighted fast spin echo sequence with the parameters of TR = 720 ms, TE = 11 ms, flip angle = 90°, image matrix = 128 × 128, field of view = 4 × 4 cm^2^, and slice thickness = 1 mm.

The signal intensity of HCC tumor, hepatic parenchyma, and background were measured by a quantification tool packaged in Preclinical Scan software. The signal‐to‐noise ratio (SNR) of tumor and liver tissue was calculated to qualify the signal enhancement efficiency in the region of interest (ROI) by the following equation: SNR = SI/σ, where SI stands for signal intensity in ROIs and σ stands for the standard deviation analyzed from the background of MR images. The sensitivity of HCC detection was determined based on the difference in signal intensity between the tumor and normal liver, i.e., the contrast‐to‐noise ratio defined as CNR = |SI_Tumor_ − SI_Liver_|/σ. The distance required to reach half of the CNR maximum from the CNR minimum location (defined as L_1/2_) was adopted to quantitatively evaluate the clarity of the tumor boundary.

### SPECT‐CT Imaging

Nude mice bearing orthotopic HCCs were intravenously injected with freshly prepared ^99m^Tc‐labeled PUSIONPs at a dose of 0.1 mmol Fe per kg body weight. SPECT‐CT images were acquired with a U‐SPECT+/CT from MILabs equipped with an extra ultra‐high sensitivity collimator (54 pinholes, reconstructed resolution 1.0 mm, sensitivity >12 500 cps/MBq) at different time points post‐injection. Anesthesia was maintained with 1.5% isoflurane during the imaging experiments. The SPECT‐CT images were reconstructed by the software package provided by MILabs. Quantification was performed by selecting the volume of interest (VOI) of the desired organs using the quantification tool of the PMOD software.

### Prussian Blue Staining

Nude mice bearing orthotopic HCC were intravenously injected with PUSIONPs at a dose of 0.1 mmol Fe per kg body weight and sacrificed at different time points, e.g., 4, 8, and 24 h, post‐injection. The anterior caudate lobe of the liver and tumor were removed, sectioned and preserved in 4% paraformaldehyde for further Prussian blue staining. The mice treated with saline were used as the control group.

### Synthesis of PUSIONPs‐FITC

First, 0.6 mg of 2‐aminoethanethiol and 7.2 mg of Tris(2‐carboxyethyl)phosphine (TCEP) were dispersed in 1 mL aqueous solution and shaken for ≈5 min. After the pH of the solution was adjusted to neutral by 1 m HEPES buffer, 0.5 mL aqueous solution containing 5 mg PUSIONPs‐mal was added and oscillated at room temperature for 4 h. The resultant amino‐functionalized PUSIONPs were purified with Millipore YM‐30 for three cycles to remove free 2‐aminoethanethiol. Then, the amino‐functionalized PUSIONPs were redispersed in deionized water to react with FITC following the protocol provided by the manufacturer. The solution was dialyzed against pure water for 72 h to remove free FITC after the reaction. Finally, the resulting PUSIONPs‐FITC solution was concentrated through ultrafiltration using Millipore YM‐30.

### FITC Labeling Stability Assessment

To evaluate the labeling stability of FITC, the as‐prepared PUSIONPs‐FITC was incubated in water, simulated body fluid (SBF), and phagolysosomal simulant fluid (PSF). Then, 10 µL of the incubation solution was extracted and mixed with 2 mL of water. After being concentrated to below 100 µL using a 30 kDa MWCO centrifugal filter, the fluorescence intensity of the filtrate was measured and compared with the total fluorescence to determine the labeling stability.

### Immunofluorescence

Mice bearing orthotopic HCC were intravenously injected with PUSIONPs‐FITC at a dose of 0.1 mmol Fe per kg body weight and then sacrificed at different time points (4, 8, and 24 h) post‐injection. The tumor and the anterior caudate lobe of the liver were harvested and preserved in 4% paraformaldehyde at 4 °C for 2 d. Then, the tissues were embedded in OCT compound, frozen on dry ice, and sectioned into 5 µm thick slices. The liver slices were immunostained with primary antibodies against anti‐F4/80 rabbit pAb for KCs (Servicebio), anti‐IYVE1 rabbit pAb for LSECs (Servicebio), anti‐CD49b rabbit pAb for LGLs (Abways), and anti‐GFAP mouse mAb for HSCs (Servicebio) at 4 °C overnight, while the tumor slices were immunostained with primary antibodies against anti‐F4/80 rabbit pAb for TAMs and anti‐IYVE1 rabbit pAb for ECs at 4 °C overnight. After that, the slices obtained were incubated with the following secondary antibodies: Cy3‐labeled goat anti‐rabbit IgG (Servicebio) and Cy3‐labeled goat anti‐mouse IgG (Servicebio) for 1 h at room temperature. For nuclear staining, DAPI (4′,6‐diamidino‐2‐phenylindole, Servicebio) was used. Under a confocal microscope, the cells were stained green by PUSIONPs‐FITC and red by antibodies labeled with Cy3.

### Liver and Tumor Fractionation

To perfuse the liver, a catheter was inserted into the portal vein and secured. The inferior vena cava was isolated and cut to allow outflow of blood and prevent pressure damage to the liver. The liver was first perfused with 50 mL of warm Ca^2+^‐ and Mg^2+^‐free Hank's balanced salt solution (HBSS) with EDTA at a rate of 4 mL mi^−1^n. The solution was then changed to 0.02% collagenase type IV in HBSS, and an additional 50 mL was perfused through the liver. During perfusion, the liver color changed from dark to pale brown after all blood was removed. For fractionation, the tumor tissue was cut and digested with collagenase solution for half an hour.

The perfused liver and tumor were placed in a sterile petri dish and agitated to release cells into solution. The cell suspensions were filtered through sterile gauze to remove undigested and connective tissues. The purified suspensions were retained as the “total liver homogenate” and “total tumor homogenate”. Cell counts were determined using a Vi‐CELL Cell Viability Analyzer. The “total liver homogenate” and “total tumor homogenate” were subsequently stained for flow cytometry analysis.

### Flow Cytometry

By pre‐incubating cells with 0.25 µg of TruStain FcX PLUS (anti‐mouse CD16/32, Biolegend) antibody per 10^6^ cells in a 100 µL volume for 10 min on ice, Fc receptors were blocked. Cells from total liver homogenate were stained with fluorophore‐conjugated monoclonal antibodies to identify the following cells: anti‐F4/80‐Brilliant Violet 421 for KCs (Biolegend), anti‐CD146‐PE for LSECs (Biolegend), anti‐CD49b‐PE for LGLs (Biolegend), and anti‐GFAP‐Alexa Fluor 647 for HSCs (Invitrogen). For intracellular staining with anti‐GFAP‐AF647 and AF647 rat IgG2a, cells were fixed and then permeabilized for 15 min with FMS‐FP0050 (Fcmacs Biotech Co., Ltd.) at room temperature. Meanwhile, cells from total tumor homogenate were stained with fluorophore‐conjugated monoclonal antibodies to identify cells through the following cell surface biomarkers, e.g., anti‐F4/80‐Brilliant Violet 421 for TAMs and anti‐CD146‐PE for ECs. The following antibodies were chosen as isotype controls: Brilliant Violet 421 Rat IgG2a (Biolegend), PE Rat IgG2a (Biolegend), PE Rat IgM (Biolegend), and Alexa Fluor 647 Rat IgG2a (Invitrogen). The cells were stained on ice for 20 min in the dark and washed twice with cell staining buffer.

Representative flow plots were acquired with FACSVerse (BD, US) and analyzed with FlowJo software (Tree Star, Inc.). The internalization efficiency of PUSIONPs into different cells was assessed by the percentage of PUSIONPs‐positive (NP^+^) cells.

### Biological TEM and STEM

Liver tissues were isolated from mice and sectioned into 2 mm × 2 mm × 2 mm blocks at different time points (4 h, 8 h, and 24 h) after PUSIONPs injection at a dose of 0.1 mmol Fe per kg body weight. Then, the blocks were fixed in 2% glutaraldehyde in 0.1 m sodium cacodylate buffer and sectioned into 100 nm thick slices. The slices were stained with uranyl acetate.

### Preparation of PUSIONPs‐Containing Liposomes

PUSIONPs‐containing liposomes were prepared as follows: 0.20 g of soybean phosphatidylcholine and 0.02 g of cholesterol were first mixed in 2 mL of diethyl ether. The resulting solution was stirred and heated to 60 °C and maintained at this temperature. Then, an ether solution containing phospholipids and cholesterol was added dropwise into the PUSIONPs solution under stirring for 2 h to completely volatilize the ether. After that, the resultant solution was centrifuged and washed with PBS buffer to collect the PUSIONPs‐containing liposomes and remove the unencapsulated PUSIONPs. By adjusting the initial PUSIONPs concentration in PBS buffer, PUSIONPs‐containing liposomes with different [Fe]_liposome_ were obtained.

### HCC Patient Population and Data Collection

In this prospective study, MRI image sequences collected between July 25, 2023, and August 30, 2023, were evaluated. The study design and procedures were previously approved by the Ethics Committee of The Fifth Affiliated Hospital, Sun Yat‐sen University, and patients provided written consent for the subsequent use of their data for research purposes. The approval number is 2023K189‐1. The inclusion criteria encompassed patients aged between 18 and 90 years with histologically or clinically confirmed diagnoses of HCC, without any prior treatments, and who had undergone T1 mapping sequence and T2 mapping sequence imaging. The exclusion criteria included patients outside the established age limits, contraindications for MRI, and a lack of T1 mapping sequence and T2 mapping sequence imaging.

### MRI Acquisition of HCC Patients

Patients underwent multiparameter MRI, including T1 mapping sequence and T2 mapping sequence, before receiving any treatments. MR imaging was conducted using a 3.0 T MRI system (Magnetom Prisma, Siemens Healthcare, Erlangen, Germany). T1 mapping sequence and T2 mapping sequence were utilized to image the liver and HCC lesions in detail prior to Primovist‐enhanced MRI. These sequences were matched at the same level, particularly at the level of the largest tumor surface area in the transverse plane. The slice thickness was set to 8 mm. T1 mapping sequence and T2 mapping sequence scans were performed during a deep inspiration phase, with a 15 s breath‐hold command. T1 and T2 values (in milliseconds) for lesions and liver parenchyma were obtained by manually drawing two ROIs of 0.25 cm^2^ while avoiding large blood vessels to ensure reliable results. The data are reported as the mean ± standard deviation.

### Statistical Analysis

Data are presented as the mean ± standard deviation (SD). For normally distributed data sets with equal variances, unpaired Student's *t*‐test was carried out across groups. Statistical significance was represented in graphs using the following convention: ***p < 0.001, **p < 0.01, *p ≤ 0.05, ns = not significant (P > 0.05). In all cases, significance was defined as *p* ≤ 0.05. Statistical analysis was carried out using Excel Software.

### Calculation Section

Details are provided in the Supporting Information Appendix.^[^ ^28^, ^35^ ^]^

## Conflict of Interest

The authors declare no conflict of interest.

## Supporting information

Supporting Information

## Data Availability

The data that support the findings of this study are available from the corresponding author upon reasonable request.
