# Supplementary material for: Trapped in Endosome PEGylated Ultra‐Small Iron Oxide Nanoparticles Enable Extraordinarily High MR Imaging Contrast for Hepatocellular Carcinomas
Source: Adv Sci (Weinh). 2024 Aug 20;11(39):2401351. doi: 10.1002/advs.202401351 (PMC11497028; doi:10.1002/advs.202401351)
Supplement: Supplementary file 1 — Supporting Information [file ADVS-11-2401351-s001.pdf]

ADVANCED SCIENCE
Open Access

Supporting Information

for *Adv. Sci.*, DOI 10.1002/advs.202401351

Trapped in Endosome PEGylated Ultra-Small Iron Oxide Nanoparticles Enable Extraordinarily High MR Imaging Contrast for Hepatocellular Carcinomas

Dandan Zhou, Shanshan Shan, Lei Chen, Cang Li, Hongzhao Wang, Kuan Lu, Jianxian Ge, Ning Wang, Mohammad Javad Afshari, Yaqin Zhang, Jianfeng Zeng* and Mingyuan Gao**

Supporting Information

Trapped in Endosome PEGylated Ultra-Small Iron Oxide Nanoparticles Enable Extraordinarily High MR Imaging Contrast for Hepatocellular Carcinomas

Dandan Zhou, Shanshan Shan, Lei Chen, Cang Li, Hongzhao Wang, Kuan Lu, Jianxian Ge, Ning Wang, Mohammad Javad Afshari, Yaqin Zhang, Jianfeng Zeng,* and Mingyuan Gao,**

Calculation Section

<u>Quantitative estimation of the concentration of PUSIONPs in the hepatic parenchyma</u>

The liver is a blood-rich organ composed of hepatic parenchyma and liver vessels, *i.e.*, the hepatic artery and portal triads[37]. To quantitatively estimate the concentration of PUSIONPs in the hepatic parenchyma, the nanoparticles in the blood vessels should be excluded. The blood circulation behavior of PUSIONPs shown in Figure S5 can be fitted by a two-compartment model, and the temporal PUSIONPs concentration in the blood (C_B) can then be given by

$$C_{\mathrm{B}} = C_0 \times (0.1585 + 0.0222\mathrm{e}^{-1.493t} + 0.8503\mathrm{e}^{-0.3338t}) \quad \text{(S1)}$$

where C_0 is the initial concentration of PUSIONPs in the blood just post-injection, and t is the time point post-injection.

Generally, the total blood volume is approximately 72 mL per kg mouse[46, 47]. The initial PUSIONPs concentration in the blood can be calculated by

$$C_0 = \frac{0.1\times m}{0.072\times m} = 1.39\ \mathrm{mM} \quad \text{(S2)}$$

for a mouse with a body weight of m kg after being injected with PUSIONPs at a dose of 0.1 mmol Fe/kg body weight.

Thus, the temporal blood concentration of PUSIONPs can be expressed by

$$C_{\mathrm{B}} = 1.39 \times (0.1585 + 0.0222\mathrm{e}^{-1.493t} + 0.8503\mathrm{e}^{-0.3338t}) \quad \text{(S3)}$$

Assuming that PUSIONPs in the liver are mainly distributed in the hepatic parenchyma and blood, we can obtain the following expression for the particle concentration in the hepatic parenchyma:

$$C_{\mathrm{HP}} = (C_{\mathrm{L}} \times V_{\mathrm{L}} - C_B \times V_{LV})/V_{\mathrm{HP}} \quad \text{(S4)}$$

where C_L is the average PUSIONPs concentration in the liver, which can be experimentally determined by SPECT, V_L is the volume of liver, V_{HP} is the volume of hepatic parenchyma, and V_{LV} is the volume of liver vessels. It has been reported that the hepatic parenchyma and liver vessels account for 93% and 7% of the total volume of the liver, respectively[48]. Thus, C_{HP} can be expressed as follows:

$$C_{\mathrm{HP}} = \frac{C_{\mathrm{L}} - 7\% \times C_{\mathrm{B}}}{93\%} \quad \text{(S5)}$$

By combining Eq. S3 and S5, the PUSIONPs concentration in the hepatic parenchyma can then be obtained.

Quantitative estimation of the concentration of PUSIONPs in liver cells

Since the extracellular spaces are directly connected to the blood, the concentration of PUSIONPs in these spaces can be considered to be the same as that in blood, *i.e.*,

$$C_{\mathrm{LES}} = C_{\mathrm{B}} \quad \text{(S6)}$$

where C_{LES} is the concentration of PUSIONPs in the liver extracellular space. Then, the concentration of PUSIONPs in liver cells in the hepatic parenchyma can be obtained by

$$C_{\mathrm{LC}} = (C_{\mathrm{HP}} \times V_{\mathrm{HP}} - C_{\mathrm{LES}} \times V_{\mathrm{LES}})/V_{\mathrm{LC}} \quad \text{(S7)}$$

where C_{LC} is the average concentration of PUSIONPs in liver cells and V_{LC} and V_{LES} are the volume of liver cells and liver extracellular space, respectively. According to a previous report, liver cells account for 84% of the hepatic parenchyma volume, and liver extracellular spaces account for 16%[49]. Thus, the concentration of PUSIONPs in liver cells can be expressed by

$$C_{\mathrm{LC}} = \frac{C_{\mathrm{HP}} - 16\% \times C_{\mathrm{B}}}{84\%} \quad \text{(S8)}$$

Quantitative estimation of the concentration of PUSIONPs in endosomes of liver cells

As demonstrated through TEM studies, PUSIONPs mainly accumulate in the endosomes of various kinds of liver cells. The volume of endosomes occupied by PUSIONPs can be obtained by summing the occupied endosome volumes of different liver cells, *i.e.*,

$$V_{\mathrm{endosome}} = \sum(R_{\mathrm{NP+}} \times V_{\mathrm{HP}} \times F_{\mathrm{VC}}) \times F_{\mathrm{VL}} \quad \text{(S9)}$$

where $R_{\mathrm{NP+}}$ represents the ratio of each PUSIONPs-positive liver cell, F_{VC} is the volume fraction for each kind of liver cell in the hepatic parenchyma, and F_{VL} is the volume fraction of endosomes in the hepatic parenchyma, *i.e.*, approximately 1.46%[50]. The concentration of PUSIONPs in endosomes can be expressed by

$$[Fe]_{\mathrm{endosome}} = C_{\mathrm{LC}} \times V_{\mathrm{LC}} / V_{\mathrm{endosome}} \quad \text{(S10)}$$

Combining Eq. S8-10, we can calculate $[Fe]_{\mathrm{endosome}}$ by

$$[Fe]_{\mathrm{endosome}} = \frac{84\% \times C_{\mathrm{LC}}}{\sum(R_{\mathrm{NP+}} \times F_{\mathrm{VC}}) \times F_{\mathrm{VL}}} \quad \text{(S11)}$$

In Eq. S11, $R_{\mathrm{NP+}}$ can be determined by flow cytometry analysis, and F_{VC} can be found in the literature and is shown in Table S4[47], thus the concentration of PUSIONPs in endosomes can be quantitatively estimated.

Calculation of the relaxivity of PUSIONPs-in-liposomes

As shown in Table S3, regardless of the $[\mathrm{Fe}]_{\mathrm{liposome}}$, the r_1 of PUSIONPs-containing liposomes is approximately a constant value, *i.e.*, 1.5 $\mathrm{mM^{-1}s^{-1}}$, while r_2 exhibits a linear relationship with the $[\mathrm{Fe}]_{\mathrm{liposome}}$, which can be expressed as

$$r_2 = 1.5702 \times [Fe]_{\mathrm{liposome}} + 39.741 \quad \text{(S12)}$$

Similarly, the r_2 of PUSIONPs-containing endosomes can be calculated once the $[Fe]_{\mathrm{endosome}}$ in the tissue is given.

Calculation of SEE of hepatic parenchyma

As 84% of hepatic parenchyma is taken by liver cells by volume and the remaining 16% by extracellular spaces, the SEE of hepatic parenchyma can be expressed by

$$SSE_{\mathrm{LV}} = 84\% \times SSE_{\mathrm{LC}} + 16\% \times SSE_{\mathrm{LES}} \quad \text{(S13)}$$

where SEE_{LV}, SEE_{LC} and SEE_{LES} are SEE for hepatic parenchyma, liver cells, and liver extracellular spaces, respectively. Both SEE_{LC} and SEE_{LES} can be calculated with Eq. 2 in the main manuscript. For the calculation of SEE_{LC}, 1.5 mM^{-1} s^{-1} is taken for r_1, while r_2 is obtained by Eq. S12. Assuming that PUSIONPs are evenly distributed in the liver, the C_{LC} obtained by Eq. S8 was used instead of $[Fe]_{liposome}$. For the calculation of SEE_{LES}, r_1 and r_2 of mother PUSIONPs, *i.e.*, 7.9 mM^{-1} s^{-1} and 36.7 mM^{-1} s^{-1}, were used. In addition, C_{LES} is assumed to be equal to C_B, which can be obtained with Eq. S3.

Quantitative estimation of the concentration of PUSIONPs in the tumor matrix

Since tumor is mainly composed of tumorous tissue and blood, we can estimate the concentration of PUSIONPs in the tumor matrix by

$$C_{\mathrm{TM}} = (C_{\mathrm{T}} \times V_{\mathrm{T}} - C_{\mathrm{B}} \times V_{\mathrm{TB}})/V_{\mathrm{TM}} \quad \text{(S14)}$$

where C_{TM} is the concentration of PUSIONPs in the tumor matrix, C_T is the average concentration of PUSIONPs in the tumor site, V_T is the volume of the whole tumor, V_{TM} is the volume of the tumor matrix, and V_{TB} is the volume of blood in the tumor.

Due to the slow uptake of nanoparticles by tumor, we assume that PUSIONPs remain populated in the blood immediately after they reach the tumor site. Thus, the amount of PUSIONPs in blood is roughly equal to that in tumor at a very early stage postinjection, *e.g.*, 0.1 h. Then, the blood volume ratio in tumor can be expressed by

$$V_{\mathrm{TB}}/V_{\mathrm{T}} = C_{\mathrm{T\,(0.1\,h)}}/C_{\mathrm{B\,(0.1\,h)}} \quad \text{(S15)}$$

where $C_{T\,(0.1\,h)}$ and $C_{B\,(0.1\,h)}$ are the concentrations of PUSIONPs in the tumor and blood 0.1 h postinjection of PUSIONPs, respectively. $C_{T\,(0.1\,h)}$ was obtained according to the SPECT results in Figure 3d, while $C_{B\,(0.1\,h)}$ can be calculated with Eq. S3. If the tumor blood volume ratio is defined as $F_{TB} = V_{TB}/V_T$, the concentration of PUSIONPs in the tumor matrix can further be expressed by

$$C_{\mathrm{TM}} = \frac{C_{\mathrm{T}} - F_{\mathrm{TB}} \times C_{\mathrm{B}}}{1 - F_{\mathrm{TB}}} \quad \text{(S16)}$$

Calculation of SEE of tumor

Similar to the calculation of SEE for the hepatic parenchyma, the SEE of the tumor can be expressed by

$$SSE_{\mathrm{T}} = (1 - F_{\mathrm{TB}}) \times SSE_{\mathrm{TM}} + F_{\mathrm{TB}} \times SSE_{B} \quad \text{(S17)}$$

where SEE_T, SEE_{TM} and SEE_B are the SEE of the tumor, tumor matrix, and tumor blood, respectively. Both SEE_{TM} and SEE_B can be calculated with Eq. 2 in the body-text. For the calculation of SEE_{TM}, 1.5 mM^{-1} s^{-1} is taken for r_1, while r_2 is obtained by Eq. S12. Assuming that PUSIONPs are mainly populated in the endosomes of tumor cells and that the concentration of PUSIONPs in endosomes is 100 times higher than that in the tumor matrix, *i.e.*, $[Fe]_{endosome} = 100 \times C_{TM}$, C_{TM} obtained by Eq. S16 was used instead of $[Fe]_{liposome}$ to obtain SEE_{TM}. In detail, 1.5 mM^{-1} s^{-1} is taken for r_1, and r_2 is obtained by Eq. S12. For the calculation of SEE_B, r_1 and r_2 of mother PUSIONPs, *i.e.*, 7.9 mM^{-1} s^{-1} and 36.7 mM^{-1} s^{-1}, were used, and C_B was obtained with Eq. S3.

Estimation of the concentration of PUSIONPs in human liver

Due to the hydrodynamic size of PUSIONPs exceeding 10 nm, their renal clearance presents a considerable challenge, leading to their predominant accumulation within the liver. If we extend the same dosage considerations to the clinical diagnosis of HCC patients, we can assume an individual's weight as "m" kg and the liver's density as 1 g/cm³. Recognizing that the liver constitutes approximately 2% of the total body weight,[6] an assumption that the injected PUSIONPs are entirely uptake by the liver yields an average iron concentration in the liver, calculated as follows:

(0.1 * m)/(0.02 * m) = 5 mM

However, if we postulate that the proportion of PUSIONPs uptake by the liver spans from 10% to 80%, the average iron concentration within the liver could fluctuate within the range of 0.5 to 4 mM.

Estimation of the concentration of PUSIONPs in human HCC

Due to the lack of surface modifications with specific targeting moieties, PUSIONPs do not possess inherent molecular targeting abilities. Instead, their ability to reach tumor sites relies mainly on the enhanced permeability and retention (EPR) effect. However, the degree of accumulation achieved through this mechanism is typically limited. Although the extent of this effect is influenced by various factors, including size, shape, and surface modifications, in mouse models, the quantity of nanomaterial taken up by tumors through the EPR effect generally remains below 5%ID/g. Utilizing this value for calculation, for an injection dose of 0.1 mmol/kg and assuming a mouse weight of 20 g and tumor density of 1 g/cm³, the resultant average iron concentration within the tumor region would be:

0.1 * 0.02 * 5%/0.001 = 0.1 mM

Drawing insights from the literature[51], it is reasonable to conjecture that PUSIONPs uptake within mouse tumor regions typically falls within the range of 0.5-5% ID/g. If we posit that the extent of PUSIONPs uptake within actual HCC patient tumors aligns with that observed in animal models, then the average iron concentration within the tumor region could range from 0.01 to 0.1 mM.

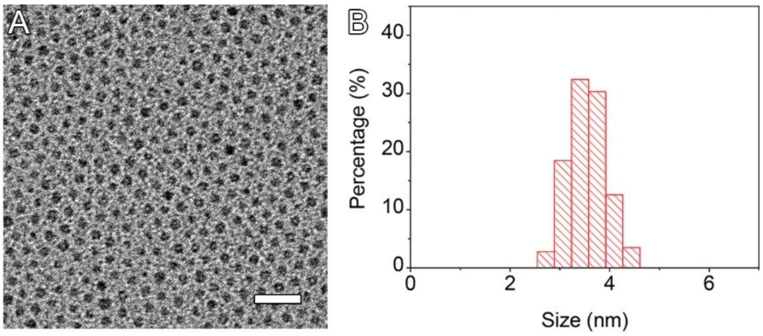

Figure S1.
(A) Representative TEM image and (B) size distribution histogram of hydrophobic ultra-small IONPs (the scale bar corresponds to 20 nm).

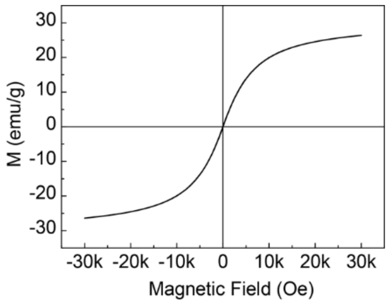

Figure S2.
The magnetization curve of PUSIONPs.

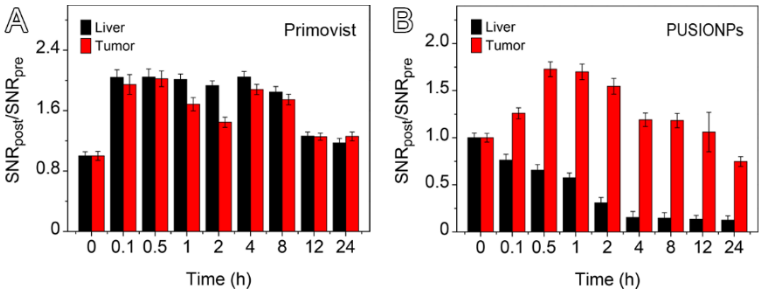

Figure S3.

(A-B) Temporal variations in the relative SNR values of the hepatic parenchyma and tumor after the intravenous administration of Primovist (A) and PUSIONPs (B), respectively, with an interval of 24 h between the two shots. Data are presented as mean ± SD. The mean represents the signal intensity in the region of interest (ROI), while the SD denotes the standard deviation calculated from the ROI of MR images.

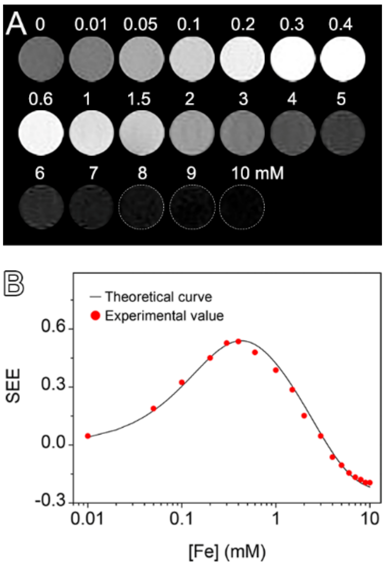

Figure S4.

(A) T1-weighted images of aqueous solutions containing PUSIONPs with different concentrations as embedded. (B) The SEE values extracted from the above images for comparison with the theoretical curve described by Equation 2.

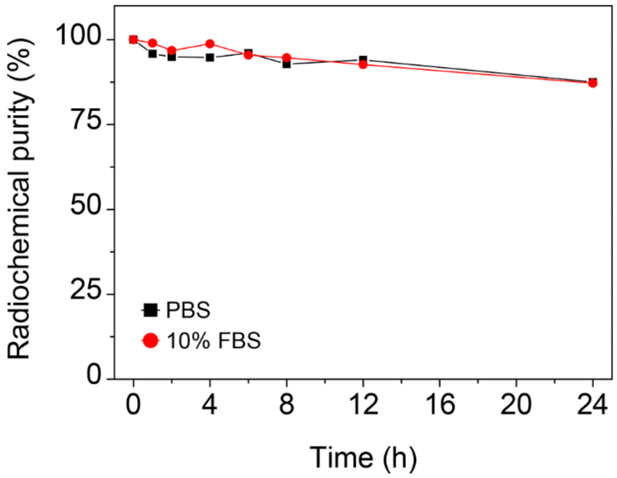

Figure S5.
Radiolabeling stability of ^{99m}Tc-labeled PUSIONPs after incubation in PBS and DMEM containing 10% FBS.

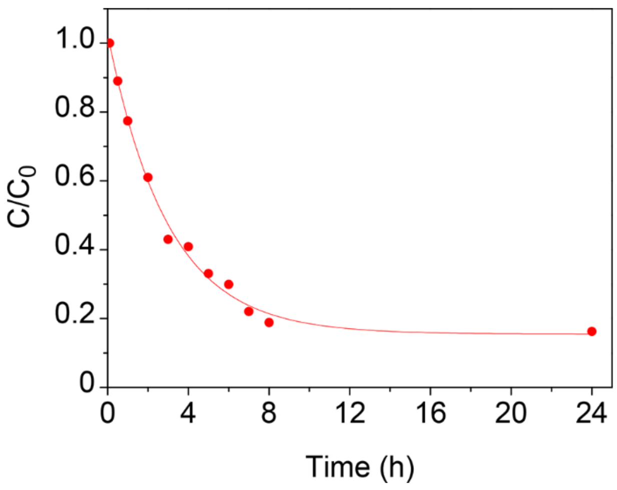

Figure S6.
Blood circulation behavior (solid line) of PUSIONPs obtained by fitting the heart signals (solid spots) with a two-compartment model.

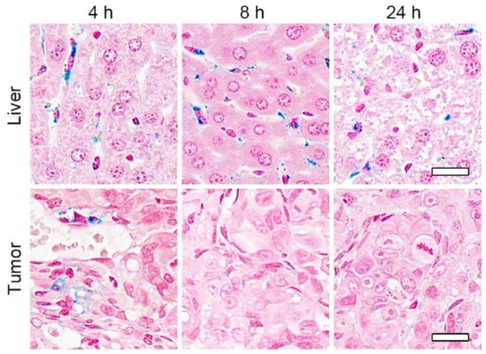

Figure S7.

Representative Prussian blue iron staining images of liver and tumor tissue extracted at different time points post intravenous injection of PUSIONPs (the scale bar corresponds to 20 μm).

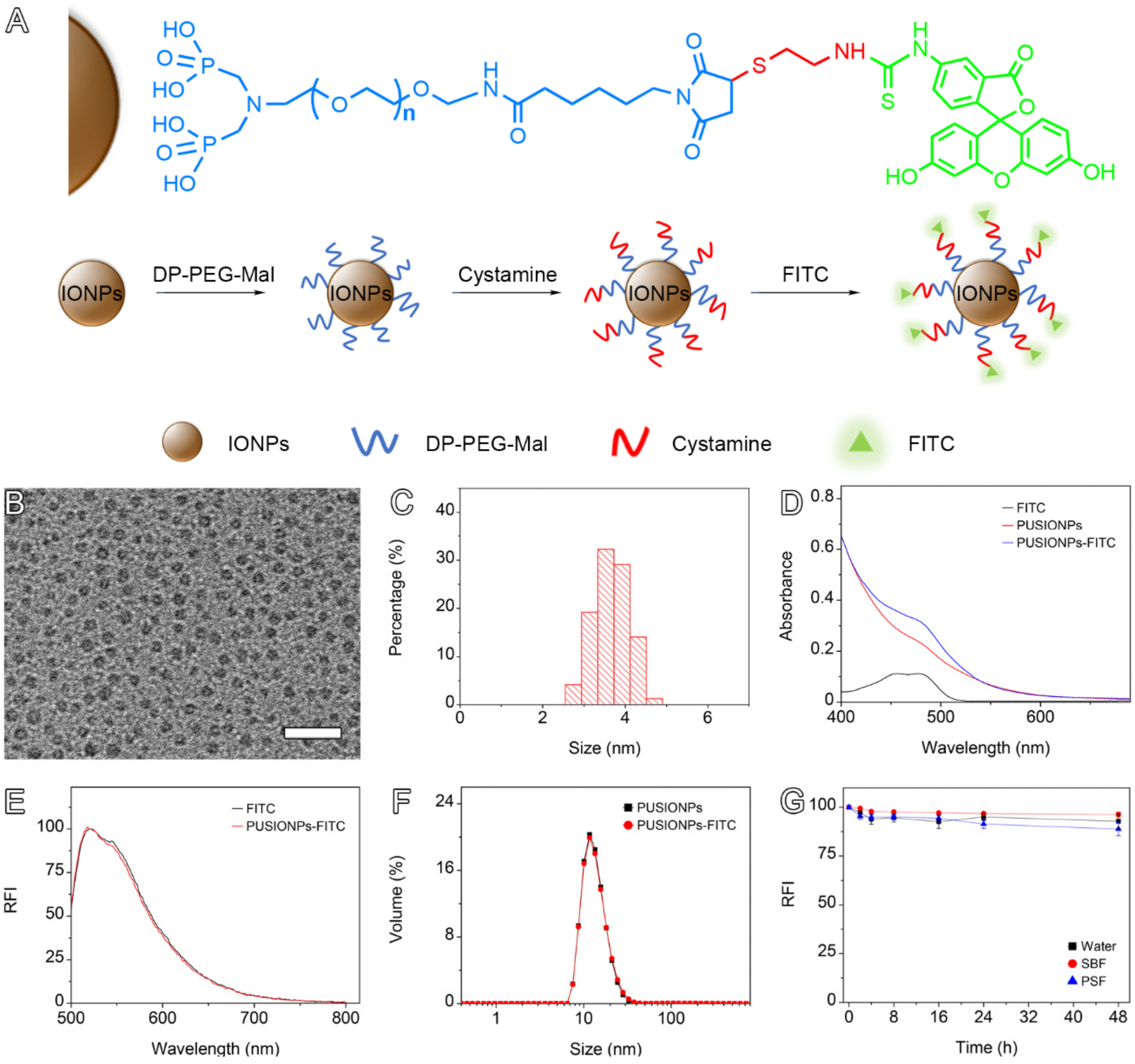

Figure S8.
(A) Schematic illustration of the synthesis of PUSIONPs-FITC. (B-C) TEM image (B) and size distribution histogram (C) of PUSIONPs-FITC (the scale bar corresponds to 20 nm). (D) UV–Vis absorption spectra of FITC, PUSIONPs, and PUSIONPs-FITC. (E) Fluorescence spectra of FITC and PUSIONPs-FITC. (F) Hydrodynamic size profiles of PUSIONPs and PUSIONPs-FITC in aqueous solution. (G) Labeling stability of PUSIONPs-FITC in water, simulated body fluid (SBF), and phagolysosomal simulant fluid (PSF). Data are presented as mean ± SD, *n* = 3.

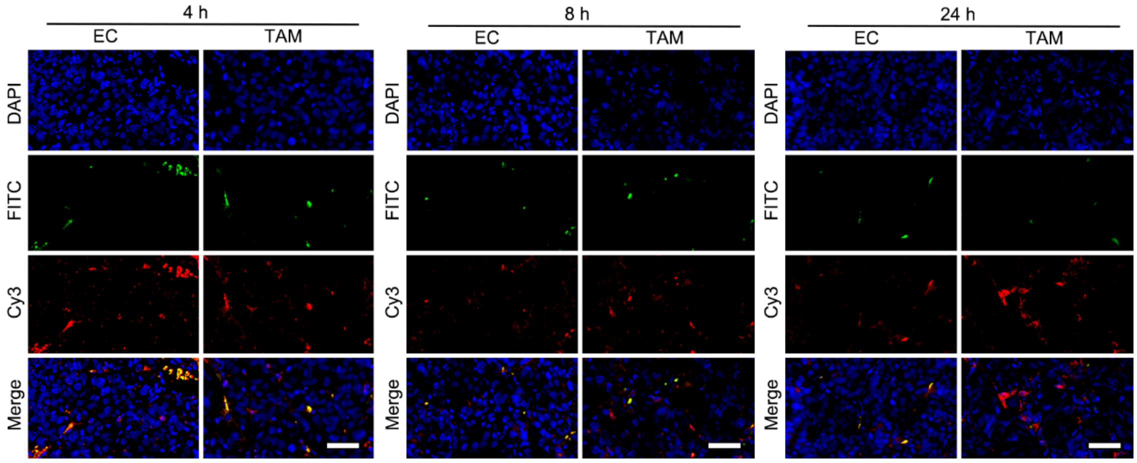

Figure S9.
Immunofluorescence images of tumor tissues extracted 4 h, 8 h, and 24 h post-injection from the tumor-bearing mice that received PUSIONPs-FITC through intravenous injection (the scale bar corresponds to 40 μm), $n = 3$.

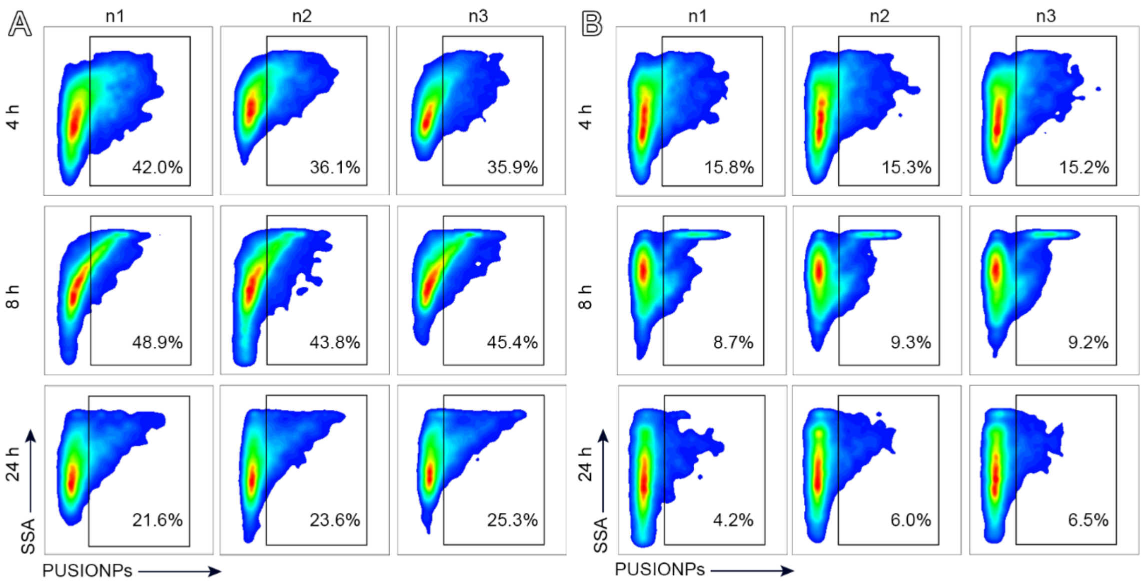

Figure S10.
Representative flow plots comparing the percentage of PUSIONPs-positive cells in the liver and tumor at different time points post-injection.

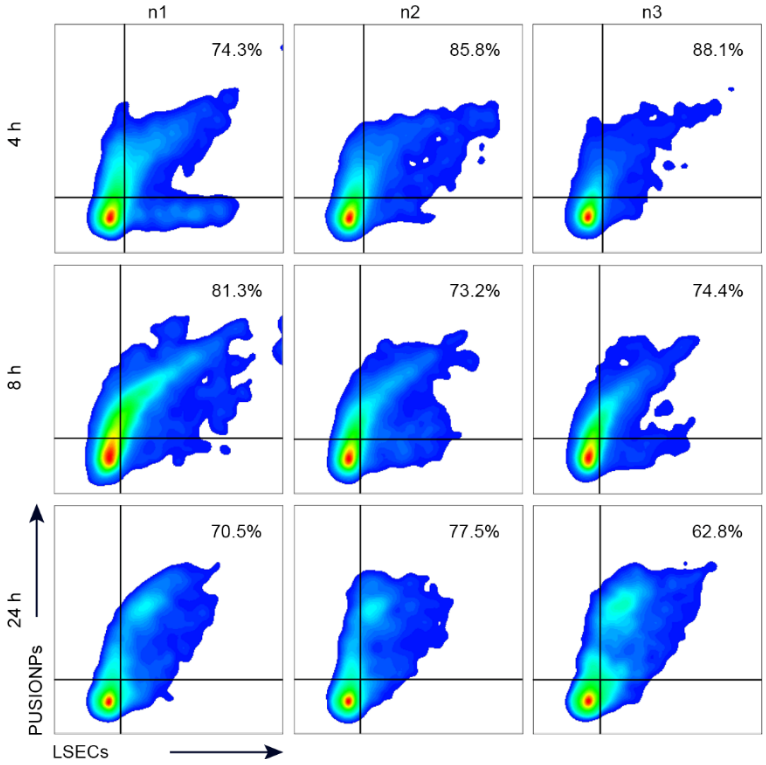

Figure S11.
Representative flow plots showing the ratio of PUSIONPs-positive cells in LSECs from the liver at different time points post-injection.

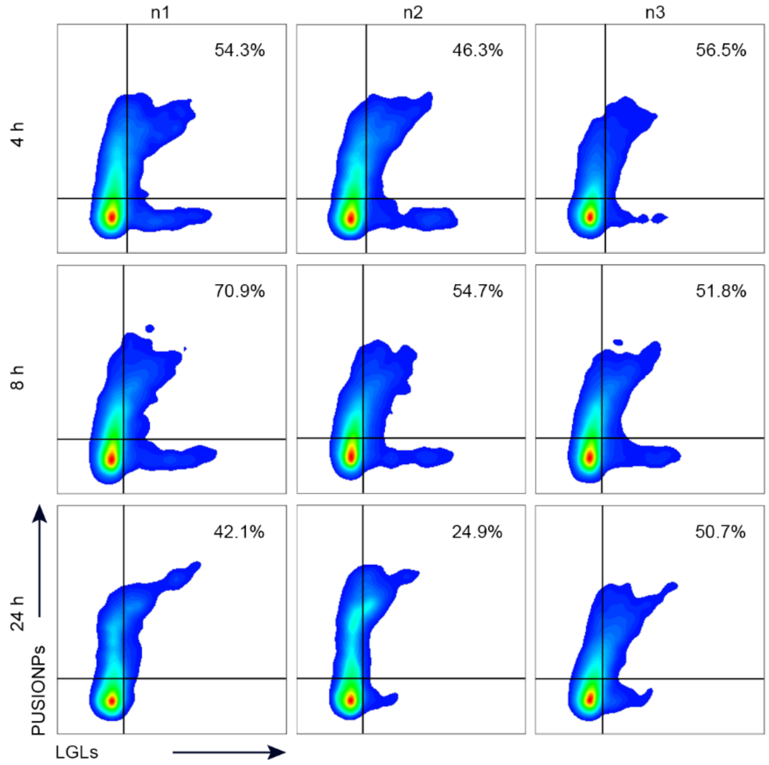

Figure S12.

Representative flow plots showing the ratio of PUSIONPs-positive cells in LGLs from the liver at different time points post-injection.

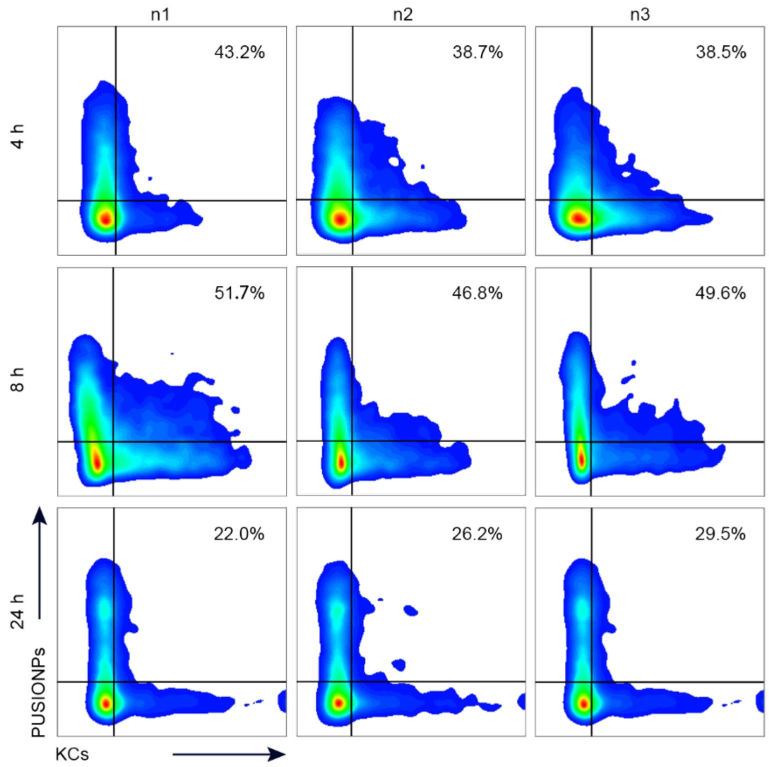

Figure S13.
Representative flow plots showing the ratio of PUSIONPs-positive cells in KCs from the liver at different time points post-injection.

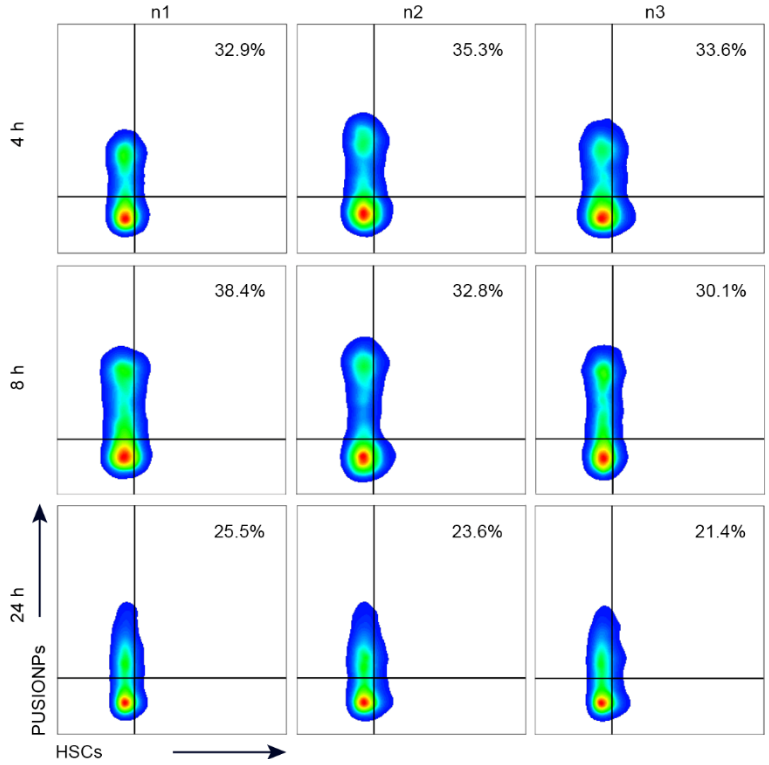

Figure S14.
Representative flow plots showing the ratio of PUSIONPs-positive cells in HSCs from the liver at different time points post-injection.

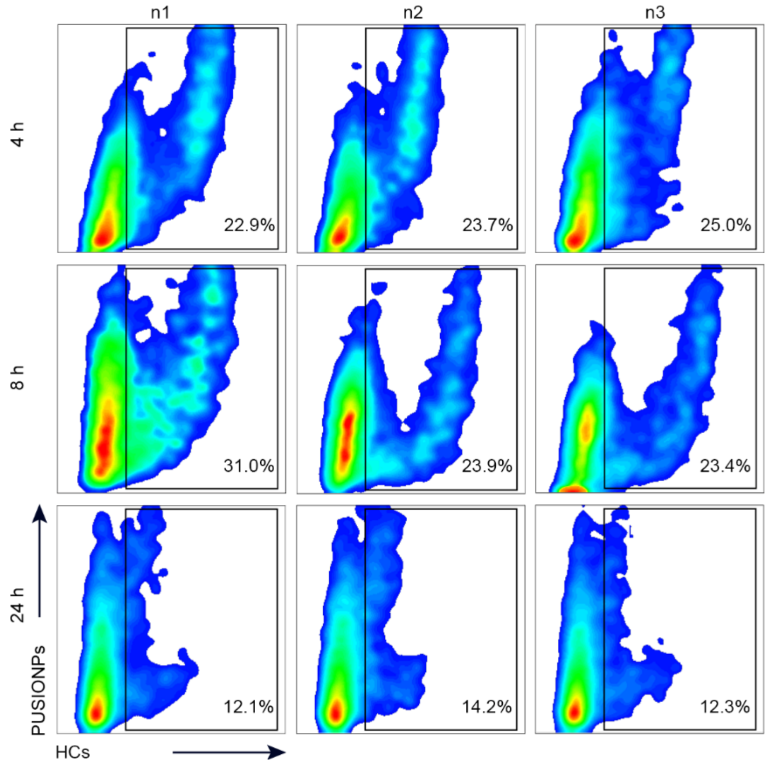

Figure S15.
Representative flow plots showing the ratio of PUSIONPs-positive cells in HCs from the liver at 4 h (A), 8 h (B), and 24 h (C) post-injection.

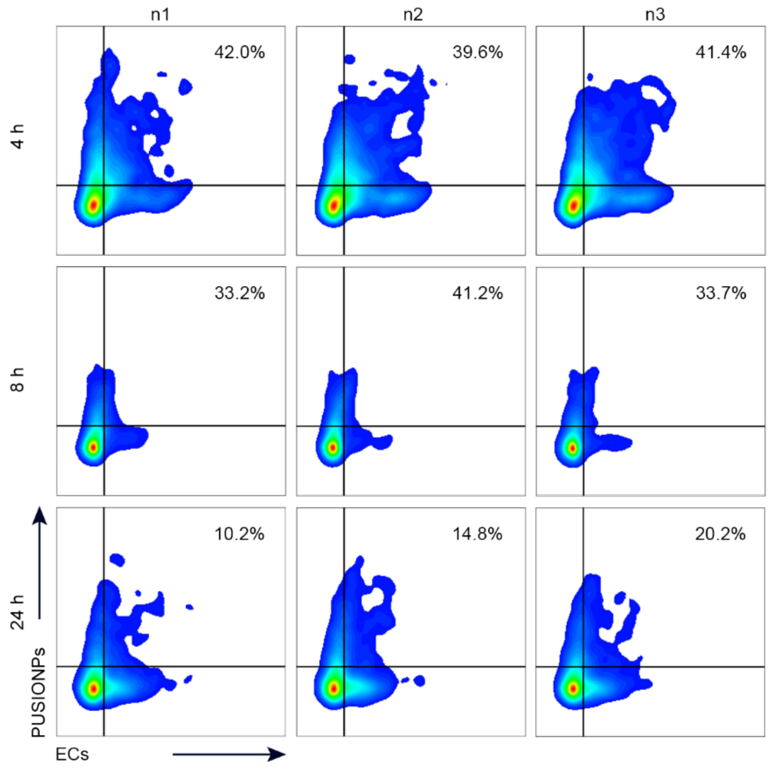

Figure S16.
Representative flow plots showing the ratio of PUSIONPs-positive cells in ECs from the tumor at different time points post-injection.

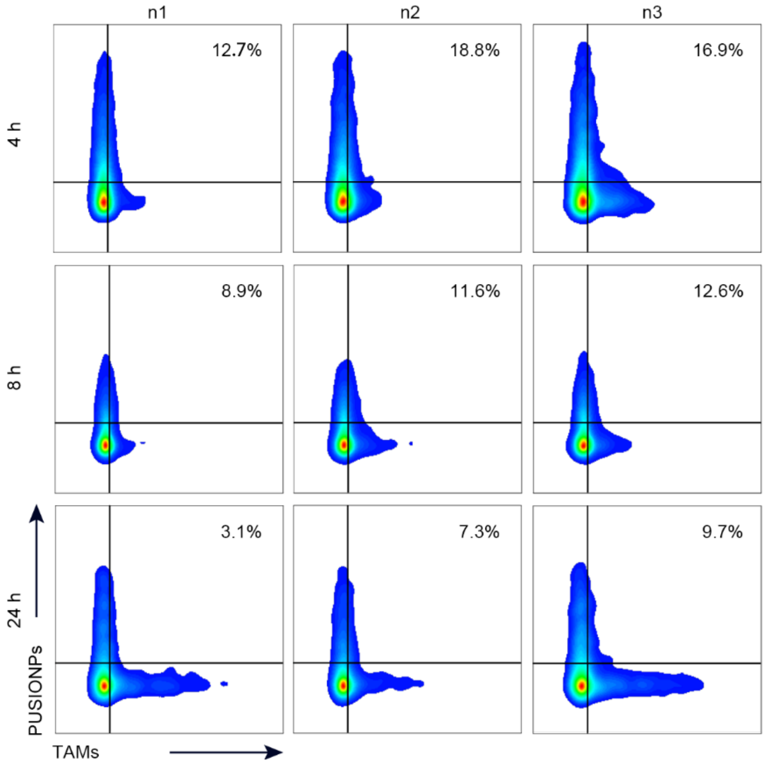

Figure S17.
Representative flow plots showing the ratio of PUSIONPs-positive cells in TAMs from the tumor at different time points post-injection.

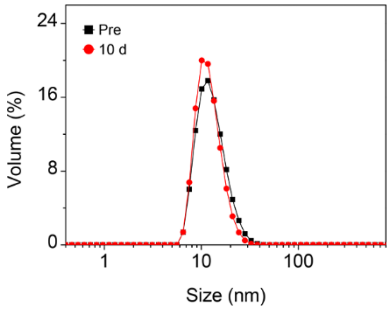

Figure S18.
Hydrodynamic size profiles of PUSIONPs before and after incubation in phagolysosomal simulant fluid (pH = 4.5) for 10 d.

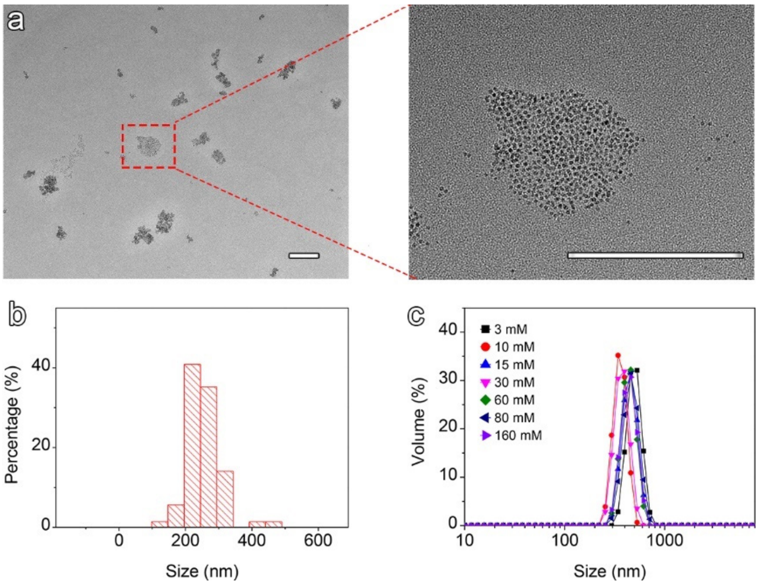

Figure S19.
(A-B) Representative TEM image (A) and size distribution histogram (B) for PUSIONPs-containing liposomes with $[Fe]_{liposome}$ of 60 mM (inset: an enlarged TEM image in the red rectangle, the scale bar corresponds to 200 nm). (C) Hydrodynamic size profiles of PUSIONPs-containing liposomes with different $[Fe]_{liposome}$.

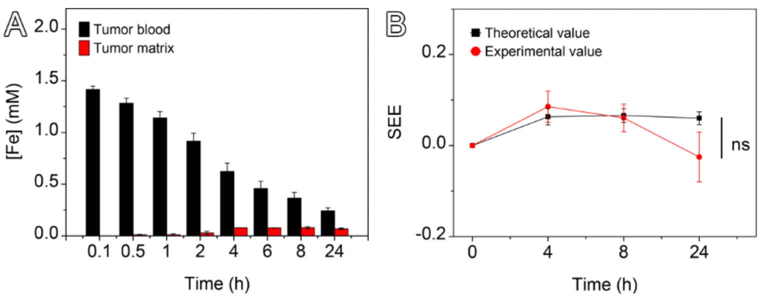

Figure S20.
(A) Iron concentration in tumor blood and tumor matrix obtained at different time points after intravenous injections of PUSIONPs. Data are presented as mean ± SD, n = 3. (B) The experimentally determined and theoretically derived temporal SEE values of the tumor site by assuming that PUSIONPs are mainly populated in the endosomes of tumor cells. Data are presented as mean ± SD, n = 3, P-values are calculated using unpaired Student's t test, ns = not significant (P > 0.05).

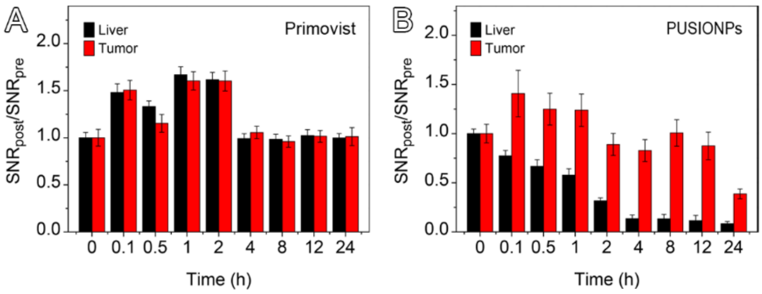

Figure S21.

(A-B) Temporal variations in the relative SNR values of the hepatic parenchyma and tumor after the intravenous administration of Primovist (A) and PUSIONPs (B), respectively. Data are presented as mean ± SD. The mean represents the signal intensity in the ROI, while the SD denotes the standard deviation calculated from the ROI of MR images.

Table S1.
Experimentally determined relaxation times of water, liver, and tumor, together with TR and TE used for MRI scanning.

Matrix	T_{1m} (s)	T_{2m} (s)	TR (s)	TE (s)
Water	2.650	0.720	0.720	0.011
Liver	0.832	0.048	0.720	0.011
Tumor	1.507	0.122	0.720	0.011

Table S2.

Pearson's correlation coefficient (PCC) for evaluating colocalization of different entities upon various fluorescence staining.

Time	Liver				Tumor	
	KC	LSEC	LGL	HSC	TAM	EC
4 h	0.26	0.71	0.68	0.75	0.70	0.72
8 h	0.45	0.56	0.58	0.89	0.49	0.68
24 h	0.21	0.40	0.50	0.87	0.20	0.72

Table S3.

The detailed STEM elemental analysis for Figure 5.

Time point post-injection	Cell type	Atomic percentage (at. %)		
		C	O	Fe
4 h	LSEC	74.17	23.31	2.52
	KC	72.70	24.52	2.78
	HSC	75.01	23.48	1.51
8 h	LSEC	77.33	20.69	1.98
	KC	76.16	21.66	2.18
	HSC	73.50	23.48	3.02
24 h	LSEC	77.82	20.63	1.55
	KC	76.58	21.05	2.37
	HSC	82.23	17.32	0.45

Table S4.

Relaxivity values of PUSIONPs-containing liposomes with different $[Fe]_{liposome}$.

$[Fe]_{liposome}$ (mM)	3	10	15	30	60	80	160
r_1 ($mM^{-1}s^{-1}$)	1.6	1.5	1.5	1.5	1.5	1.4	1.6
r_2 ($mM^{-1}s^{-1}$)	31.4	56.4	72.4	86.0	141.5	164.6	288.0

Table S5.
The volume fractions of different liver cells in the hepatic parenchyma.

Cell type	F_{VC} (%)
KC	2.1±0.31
LSEC	2.8±0.19
HSC	1.4±0.19
HC	77.8±1.15
LGL	<1

Table S6.

$L_{1/2}$ values for differently sized HCCs enhanced by Primovist and PUSIONPs.

Tumor volumes (mm^3)	15	0.3
Primovist	0.33	0.21
PUSIONPs	0.25	0.17
